# moiraine: an R package to construct reproducible pipelines for the application and comparison of multi-omics integration methods

**DOI:** 10.1093/bioinformatics/btag070

**Published:** 2026-02-15

**Authors:** Olivia Angelin-Bonnet, Lindy Guo, Roy Storey, Susan Thomson

**Affiliations:** Data Science, Bioeconomy Science Institute, Palmerston North 4442, New Zealand; Data Science, Bioeconomy Science Institute, Auckland 1142, New Zealand; Kiwifruit New Cultivars, Bioeconomy Science Institute, Te Puke 3182, New Zealand; Molecular & Digital Breeding, Bioeconomy Science Institute, Christchurch 8140, New Zealand

## Abstract

**Motivation:**

In the past decades, many statistical methods for integrating multi-omics data have been developed. They have been implemented into software tools, which differ widely in their programming choices, such as the format required for data input, or the format of the generated integration results. This lack of standards renders cumbersome and time-intensive the application and comparison of different integration tools to the same multi-omics dataset.

**Results:**

We have developed the moiraine R package for constructing reproducible multi-omics integration pipelines, which enables users to apply one or more statistical methods for multi-omics integration to their own multi-omics dataset. moiraine facilitates the preprocessing of the omics datasets and automates their formatting for the integration step. It simplifies the interpretation and evaluation of the integration results through the construction of visualizations in which metadata about samples and features can easily be included. Crucially, it enables the comparison of results obtained with different integration tools, allowing users to assess the robustness of their results.

**Availability and implementation:**

The moiraine R package is publicly available at https://github.com/Plant-Food-Research-Open/moiraine; an archival snapshot of the package is available on Zenodo at https://doi.org/10.5281/zenodo.17172718. A detailed tutorial is available at https://plant-food-research-open.github.io/moiraine-manual/.

## 1 Introduction

In the past two decades, we have witnessed an explosion in the number of multi-omics studies, in which measurements from several omics layers (e.g. genome, transcriptome, proteome or metabolome) are obtained for the same set of biological samples. This has prompted the development of statistical methods dedicated to the integrated analyses of these datasets in order to extract insights into the underlying biological processes spanning the different omics layers (Zeng and Lumley 2018, Krassowski *et al.* 2020, Subramanian *et al.* 2020, Vahabi and Michailidis 2022), many of which have been implemented into software tools or packages (Rohart *et al.* 2017, Argelaguet *et al.* 2018, el Bouhaddani *et al.* 2018, Gu *et al.* 2021, Velten *et al.* 2022). Comparison studies have highlighted the lack of a gold standard integration method, as each method performs differently depending on the biological scenario, the type of omics data, the number of samples and features, etc (Cantini *et al.* 2021, Leng *et al.* 2022, Wissel *et al.* 2023). Consequently, it is beneficial for researchers to try more than one integration method when analysing their own multi-omics datasets and to compare their results. However, the application and comparison of different integration tools are complicated by the lack of standards in their implementation (Pierre-Jean *et al.* 2020), in particular the differences in the formatting of the input data and metadata required, as well as in the format in which the results are returned.

As a result, several packages have been implemented in order to facilitate the application and comparison of different integration tools to a user-provided multi-omics dataset. A number of them, such as the CEPICS (Duan *et al.* 2019), MOVICS (Lu et al. 2021), and cirmmix (Pierre-Jean *et al.* 2020) R packages, focus on the task of cancer subtyping; others, like the Omix R package (Schneegans *et al.* 2023), are devoted to the integration of specific omics types—in the case of Omix, transcriptomics and proteomics datasets. Most of these tools provide options for data preprocessing, including normalization, transformation, and feature prefiltering, which has been shown to greatly impact the results of multi-omics integration (Amaro *et al.* 2022). Some of them also offer functionalities to interpret the integration results, although these are mostly targeted at human data and clinical outcomes specifically. Similarly, the comparison of the integration results is restricted to the problem of sample classification for cancer subtyping, which is not informative for other research domains. In addition, little thought has been given to combining the results of different methods into a consensus output—other than for the specific task of sample classification in MOVICS—which could be used to summarize the integration results and assess their robustness. Lastly, most of the visualization capabilities of these frameworks are tool-specific, without a unified approach to inspecting the results from different integration methods.

To fill this gap, we have developed the R package moiraine (Multi-Omics Integration Reproducible Analysis in R), which facilitates the application, evaluation, and comparison of different multi-omics integration tools to any multi-omics dataset. Users can construct modular, reproducible, and fully customizable pipelines that cover omics data preprocessing, integration via different tools, interpretation of integration results, as well as evaluation and comparison of the findings. Contrary to existing frameworks, moiraine is not restricted to a specific research domain and can accommodate any omics type and research question (Table 1). Some of the key innovations of moiraine include: (i) strong management of metadata (i.e. information about the biological samples and molecular features measured) coupled with extensive visualization capabilities that enable users to interrogate the data and integration results in the context of their experiment, (ii) the ability for users to extract the integration results into a standardized format, which facilitates their dissemination, along with tool- and organism-agnostic visualizations and evaluation of integration results, (iii) formal comparison of integration results, both in terms of pattern detection and feature prioritization, which has not been previously proposed, and (iv) generation of a consensus importance score to combine results from different integration tools, enabling a robust prioritization of molecular features. The modular design of moiraine ensures that as new integration tools are developed, they can easily be added to the framework, ensuring its continued relevance for the evolving field of multi-omics.

**Table 1:** Comparison of key features between moiraine and similar frameworks.

	**CEPICS** (Duan *et al.* 2019)	**MOVICS** (Lu et al. 2021)	**cirmmix** (Pierre-Jean *et al.* 2020)	**Omix** (Schneegans *et al.* 2023)	moiraine
**Scope**	Cancer subtyping (samples clustering)	Cancer subtyping (samples clustering)	Samples clustering (unsupervised methods)	Human disease	General (supervised and unsupervised methods)
**Supported data**	Any omics	Any omics, all samples must be shared	Any omics	Proteomics and transcriptomics	Any omics
**Metadata use**	Samples: survival data Features: none	Samples: survival data Features: none	None	Samples: covariates for batch effect removal Features: automatic ontology detection (human or mouse only)	Samples: included in all visualizations Features: use of custom labels, included in all visualizations
**Preprocessing**	Missing values imputation Data transformation Features preselection	Missing values imputation Features preselection	None	Data transformation Features preselection Batch correction and denoising Outlier detection Single-omics analyses	Missing values imputation Data transformation Features preselection
**Integration methods**	5 samples clustering methods: SNF, PFA, LRAcluster, iClusterBayes, PINS	10 samples clustering methods: CIMLR, iClusterBayes, MoCluster, COCA, ConsensusClustering, IntNMF, LRAcluster, NEMO, PINSPlus, SNF	13 integration methods: RGCCA, intNMF, SNF, LRACluster, PINSPlus, ConsensusClustering, MCIA, mixKernel, SGCCA, iClusterPlus, MoCluster, CIMLR, MOFA	5 integration methods: MOFA, MEFISTO, iClusterBayes, DIABLO, SMBPLS	5 integration methods: sPLS, sO2PLS, DIABLO, MOFA, MEFISTO
**Post-processing**	Samples clustering evaluation Survival analysis	Samples clustering evaluation Survival analysis Genomic features analyses Functional enrichment	Samples clustering evaluation	Functional enrichment Visualization of multi-omics networks Cell clustering analysis	Visualization of integration results Comparison with features of interest Functional enrichment Samples clustering evaluation
**Comparison of integration methods**	Comparison of samples clustering performance and running time	None	Comparison of samples clustering performance	None	Comparison of latent dimensions, features ranking and running time
**Results aggregation**	Consensus samples clustering	Consensus samples clustering	None	None	Feature’s consensus importance for identification of key biomarkers
**Standard reporting of integration results**	No	No	No	No	Yes
**Reproducibility of analyses**	Not explicitly included	Not explicitly included	Not explicitly included	Not explicitly included	Seed automatically fixed and recorded for each step of the analysis pipeline

## 2 Implementation

**Figure 1:**
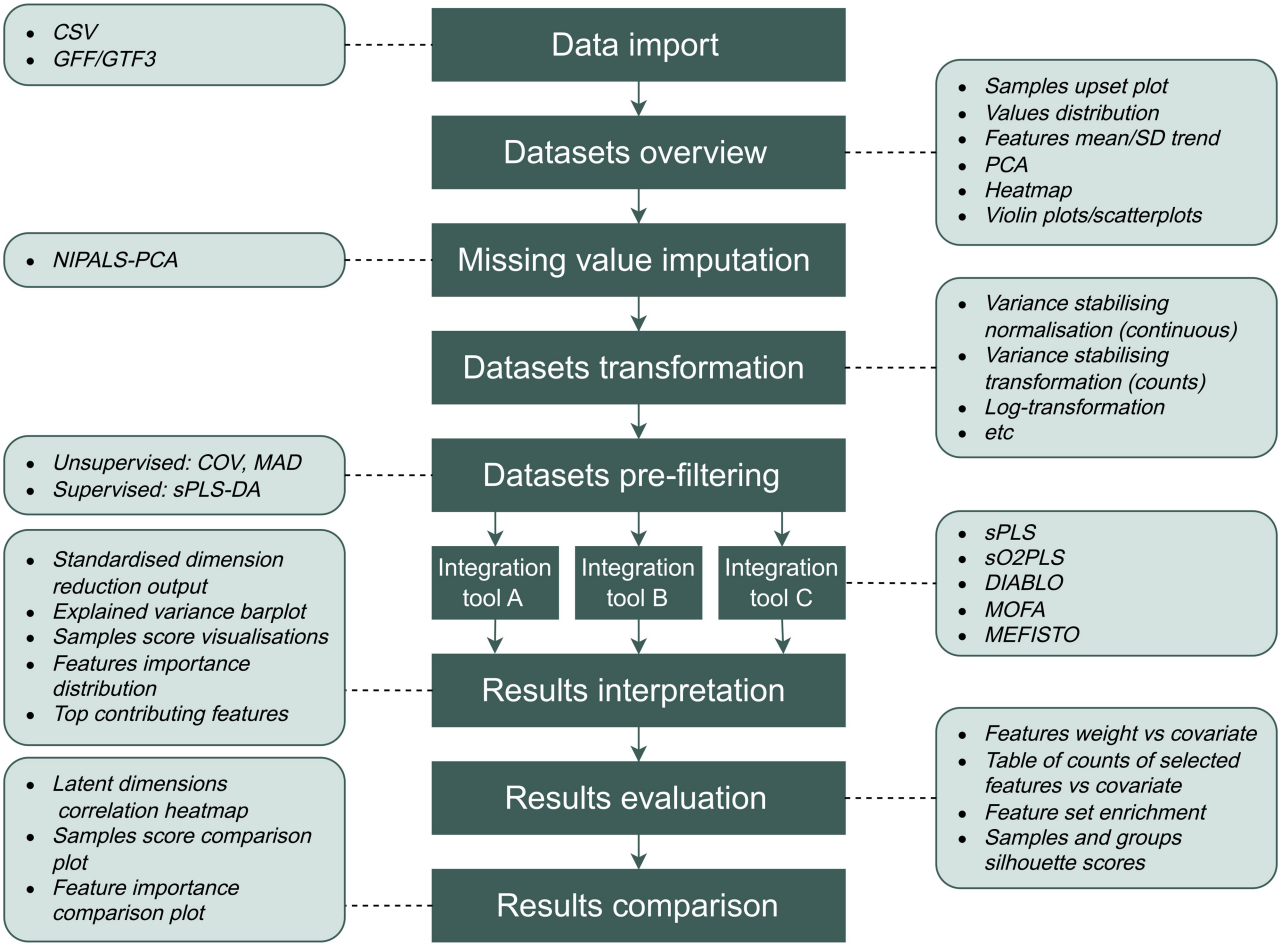
Overview of the different analysis steps in a typical multi-omics integration analysis (dark middle boxes) and the functionalities implemented in moiraine for each of these steps (light outer boxes).

In this section, we describe the capabilities of the moiraine package. Figure 1 illustrates the different steps of a typical multi-omics integration analysis spanning data import, overview, and preprocessing to integration, results interpretation and comparison, and the functionalities offered by moiraine. For some of these functionalities, moiraine builds on existing R packages, which will be mentioned throughout the section. Detailed documentation of the package is available online at https://plant-food-research-open.github.io/moiraine-manual/.

### 2.1 Data import

For each omics dataset to be integrated, the user can import a matrix of measurements, as well as information about the samples (referred to as samples metadata) such as treatment group or date of sampling, and information about the features measured (referred to as features metadata), such as gene description, compound chemical formula, etc. The package supports csv files for datasets and metadata tables, or GFF and GTF3 files for gene or transcript information.

Once imported, the omics datasets are stored alongside their samples and features metadata in a single R object; moiraine makes use of the Biobase (Huber *et al.* 2015) and MultiDataSet (Hernandez-Ferrer *et al.* 2017) packages, the latter implementing an object class specifically designed to store multiple omics datasets and associated information. This object can then be passed to the other moiraine functions for the rest of the analysis. Accessor functions allow users to easily retrieve information such as the measurement matrices or metadata tables or lists of sample and feature names.

### 2.2 Data preprocessing

#### 2.2.1 Datasets overview and missing values imputation

The package provides several visualizations and diagnostics to get an overview of the imported multi-omics data. For example, it is possible to assess the percentage of missing values in each omics dataset, to show the overlap between samples present in the different datasets with an Upset plot via the UpSetR package (Conway *et al.* 2017), or to display the relationship between the features’ means and standard deviations across samples (which is useful to decide whether data transformation is necessary).

It is also possible to visualize the measurements for specific features of interest from one or more omics datasets, either as a heatmap through the ComplexHeatmap package (Gu *et al.* 2016) or as scatterplots or violin-and-boxplots against some categorical or continuous sample covariate of interest.

In addition, a Principal Component Analysis (PCA) can be run on each dataset independently through the pcaMethods package (Stacklies *et al.* 2007). For datasets with missing values, a Nonlinear Iterative Partial Least Squares (NIPALS) PCA is applied, and the results are used to impute missing values. A complete version of the MultiDataSet object—in which missing values in each omics dataset have been imputed—is created to be used for downstream analysis. Alternatively, users can implement their own data imputation step, as the package offers an option to modify the datasets stored in a MultiDataSet object.

#### 2.2.2 Datasets transformation

Several data transformation options are implemented in order to cater for different data types. These options include: variance-stabilizing normalization implemented in the vsn package (Huber *et al.* 2002) (for continuous values e.g. LC-MS area values), variance stabilizing transformation implemented in the DESeq2 package (Love *et al.* 2014) (for RNAseq count data), as well as common normalization methods such as z-score transformation, log-transformation, or ordered quantile transformation, as implemented in the BestNormalize package (Peterson 2021). It is also possible to use the bestNormalize package to automatically select the optimal normalization method for each feature (recommended for independent phenotypic measurements). A summary table of the transformation applied to each omics dataset can be obtained for reporting purposes. Alternatively, users can perform their own transformation of the datasets.

#### 2.2.3 Datasets prefiltering

Feature prefiltering aims at reducing the dimensionality of the omics datasets by removing non-informative features. With moiraine, this can be done in an unsupervised fashion by retaining the most variable features, either based on their median absolute deviation (MAD) or their coefficient of variation (COV) score. Alternatively, it is possible to use a supervised approach that selects features most associated with a sample outcome of interest. This is done with the sparse Partial Least Squares—Discriminant Analysis (sPLS-DA) method implemented in the mixOmics package (Lê Cao *et al.* 2011). sPLS-DA uses a supervised dimension reduction technique coupled with penalization to retain features that best discriminate predefined groups of samples.

For both unsupervised and supervised approaches, prefiltering is performed independently for each omics dataset; the user can set either the number or the percentage of features that should be retained in each dataset.

### 2.3 Data integration

The package currently accommodates five multi-omics integration methods, implemented in three R packages: sPLS (Cao *et al.* 2008) and DIABLO (Singh *et al.* 2019) from the mixOmics package (Rohart *et al.* 2017), sO2PLS from the OmicsPLS package (el Bouhaddani *et al.* 2018), as well as MOFA (Argelaguet *et al.* 2018) and MEFISTO (Velten *et al.* 2022) from the MOFA2 package. A description of each method is provided in the Supplementary Material, available as supplementary data at *Bioinformatics* online; they represent a combination of targeted (DIABLO) and untargeted (others) approaches, which can handle two (sPLS, sO2PLS) or more (DIABLO, MOFA, MEFISTO) omics datasets. For each supported method, moiraine generates the necessary input data from a MultiDataSet object, which can then be passed to the corresponding functions from the method’s package. A number of helper functions facilitate the use of these methods, e.g. to provide useful default settings or to automate specific steps (such as the pairwise comparison of datasets using PLS for DIABLO). Due to the modular nature of the package, support for additional integration methods can easily be implemented.

### 2.4 Formatting and visualization of the integration results

All integration methods mentioned above perform dimension reduction by constructing latent dimensions that are weighted combinations of the features from the omics datasets. Taking advantage of this, moiraine converts the results of any of the supported integration methods into a standard dimension reduction output format, which consists of (i) a table of features’ weights or contributions to each of the latent dimensions and (ii) a table of sample scores or coordinates for each of the latent dimensions. An importance score is computed for each feature and latent dimension, as follows:


$$m_{ij}=\frac{\left|w_{ij}\right|}{\underset{i}{\max}(|w_{ij}|)}$$

where $w_{ij}$ is the weight of feature *i* for latent dimension *j*, and the maximum is computed across all features from the same omics dataset for the corresponding latent dimension. This importance score can be used as a ranking of the features’ contributions for any given latent dimension and dataset.

The standard dimension reduction output object can then be used to visualize the integration results. For example, samples can be represented in the space spanned by the latent dimensions constructed. Alternatively, their coordinates can be compared with numerical or categorical covariates recorded in the samples metadata. The distribution of feature weights, or the importance score of the top contributing features, can be shown for each latent dimension. For the latter, custom labels from the features metadata can be used to identify features instead of the identifier used in the measurement matrix (e.g. to represent transcripts using the name of the protein they encode rather than their gene ID).

### 2.5 Methods evaluation

The moiraine package facilitates the evaluation of integration results in multiple ways. It is possible to plot the distribution of feature weights against some custom feature label—e.g. the outcome of single-omics analyses, such as whether the features were found to be differentially expressed or not, or the chemical class of metabolites. This plotting capability also extends to the comparison of feature weights to continuous covariates recorded in the features metadata, e.g. to compare feature weights to their fold change from a single-omics analysis. For integration methods performing feature selection, the user can generate a table comparing the features selected to these feature labels, e.g. to assess the overlap between single- and multi-omics analysis results.

Enrichment of the latent dimensions for feature sets provided by the user, such as GO terms or KEGG pathways, can also be performed. The enrichment analysis relies on the GAGE algorithm implemented in the gage package (Luo *et al.* 2009). This facilitates the interpretation of the latent dimensions in terms of the biological processes that they represent. The feature sets used for the enrichment can be extracted from the features metadata contained in a MultiDataSet object or generated from a separate annotation file. Functions within the package facilitate the matching of these sets to the features present in the omics datasets to ensure that an appropriate background set is used for the analysis (Timmons *et al.* 2015).

Lastly, in order to evaluate sample grouping, it is possible to compute the silhouette score of individual samples to assess whether sample groups of interest (e.g. different treatment groups or genotypes) are separated by the latent dimensions computed.

### 2.6 Methods comparison

In the moiraine package, the results from two integration methods can be compared by plotting the correlation between the sample scores and/or the feature weights of each latent dimension they construct. If the methods return similar results, we expect pairs of latent dimensions from the two methods to be highly correlated, both in terms of their sample scores and their feature weights. When comparing two or more integration methods, these correlations can be visualized as a clustered heatmap to facilitate the identification of similar latent dimensions across the methods. Additionally, the relationship between the sample scores or the feature weights of any two latent dimensions can be explored further through scatter plots.

To summarize the importance given to features for latent dimensions constructed with different methods, a consensus importance score can be calculated for each feature, as follows:


$${\text{cm}}_{i}=f(m_{id_{1}},\ldots ,m_{id_{J}})$$

where $m_{id_{j}}$ is the importance score assigned to feature *i* for latent dimension $d_{j}$ from integration method *j* (with 1≤j≤J when comparing *J* integration methods), and *f* is an aggregation function. Several aggregation functions can be used, such as average, geometric or harmonic mean, L2-norm, or product, e.g.:


$$f_{\text{av}}(m_{id_{1}},\ldots ,m_{id_{J}})=\frac{1}{J}\sum_{j=1}^{J}m_{id_{j}}$$

$$f_{\text{geom}}(m_{id_{1}},\ldots ,m_{id_{J}})=\text{exp}\;\left(\frac{1}{J}\sum_{j=1}^{J}\;\text{log}\;\left(m_{id_{j}}\right)\right)$$

$$f_{L2}(m_{id_{1}},\ldots ,m_{id_{J}})=\sqrt{\sum_{j=1}^{J}m_{id_{j}}^{2}}$$

The different aggregation functions emphasize different patterns of importance across the integration results (Fig. S1, available as supplementary data at *Bioinformatics* online); e.g. the geometric mean will prioritize features with high importance scores across all methods considered (more stringent, showing the common signal detected by all integration methods), while the L2 norm will also highlight features with high importance scores in one or a few methods (more inclusive, showing signals that are detected by some but not all methods). The consensus importance score relies on the features’ importance score rather than their original weights, as the weights are not directly comparable within or between integration methods.

### 2.7 Constructing reproducible and scalable pipelines

In order to turn an analysis script into a reproducible pipeline, moiraine makes use of the Make-like pipeline R package targets (Landau 2021). The targets package enables the construction of computational pipelines in R in which each step (called a target) is linked to other steps through their input and output and which can be visualized as a directed acyclic graph (DAG). The targets package assesses the order in which the different targets must be run and monitors the code and input data files such that upon change, any target affected—and only those targets—will be flagged as out-of-date and re-evaluated. This ensures that the analysis is always up-to-date with the code and data used, mitigating errors arising from manually running a multi-step pipeline and saving computational time by only running out-of-date targets. In addition, the output of each target is saved and easily retrievable, which gives users full access to the intermediate results of the pipeline, facilitating both error diagnosis and further investigation of the results. Each target is automatically assigned a seed so that the same pipeline run on two independent machines returns the same results, even for stochastic computations such as cross-validation. The seed used to run each target is automatically recorded along with other pipeline metadata, including the running time of each target.

The targets package also enables users to scale up their pipeline through the use of distributed computing. While by default a pipeline is run sequentially in a local process, it can easily be configured to run several targets in parallel as local processes or to dispatch all or specific targets to high-performance computing systems such as SLURM or cloud computing services such as AWS. This is particularly useful when analysing large multi-omics datasets (i.e. with many omics layers or with a large number of features).

The moiraine package implements a number of target factories, which are functions that facilitate the construction of specific groups of targets for repetitive tasks, such as reading several dataset files, or running a PCA on each omics dataset. This reduces code repetition and increases readability.

### 2.8 Example datasets

The capabilities of the package are illustrated with a publicly available multi-omics dataset from a study on bovine respiratory disease in beef cattle (Li *et al.* 2022). This dataset comprises SNP dosage from an Illumina GGP Bovine 100K microarray SNP chip, raw read counts from a paired-end total RNA experiment, and metabolite abundance from NMR spectroscopy. The genomics and metabolomics datasets were retrieved from the Borealis database (DOI: 10.5683/SP3/ZETWNY), and the transcriptomics dataset from the GEO database (accession GSE217317). In order to limit the computational time required to run this example analysis, the size of the genomics dataset was reduced as follows. First, all SNPs reported as significant QTLs or eQTLs in the original study were retained. Then, 23 000 SNPs were randomly sampled from those that were not significant QTLs or eQTLs. This yielded a genomics dataset of 23 036 SNPs over 139 samples. The transcriptomics dataset was filtered to remove genes with very low read counts, resulting in a dataset of 20 335 genes over 143 samples. The metabolomics dataset, containing measurements of 55 metabolites over 139 samples, was not filtered. The results of the differential expression performed on the transcriptomics and metabolomics datasets were reproduced according to the original paper’s methods. The reduced multi-omics dataset, as well as information about the samples and features are made available as part of the moiraine package. More information about the dataset and its preparation is available in Supplementary Material, available as supplementary data at *Bioinformatics* online, and the code used to retrieve and prepare the data is available on the moiraine GitHub repository at https://github.com/Plant-Food-Research-Open/moiraine/blob/main/data-raw/example_dataset_li2022.R.

We also assessed the computational requirements of the moiraine pipeline on a second multi-omics dataset from the EATRIS-Plus project (Alonso-Andrés *et al.* 2023, ’t Hoen and de Visser 2024). It comprises observations on 127 healthy individuals, covering eight omics layers (mRNAseq, miRNAseq, proteomics, enzymatic methylation sequencing, lipidomics from two ionization modes, as well as two targeted metabolomics datasets—see Supplementary Material, available as supplementary data at *Bioinformatics* online for more information). For both analyses, we used the autometric R package (Landau 2024) (https://wlandau.github.io/autometric/) to record memory usage.

## 3 Application

We illustrate the capabilities of moiraine with a publicly available beef cattle dataset consisting of genomics, transcriptomics, and metabolomics data collected from 80 animals diagnosed with bovine respiratory disease (BRD) and 63 control animals (Li *et al.* 2022). In the study, the authors used GWAS and differential expression analyses to identify SNPs, transcripts, and metabolites associated with BRD. We show how we can use moiraine to analyse this multi-omics dataset in an integrated fashion, evaluate the integration results against those presented in the original paper, and compare the performance of several integration tools, as well as assess the impact of feature preselection on the integration results. The code used to reproduce these results is made available in File 2, available as supplementary data at *Bioinformatics* online and on GitHub at https://github.com/Plant-Food-Research-Open/moiraine_publication_code.

### 3.1 Data preparation

Using moiraine, the transcriptomics dataset was transformed from raw read counts to a log2-scale via variance-stabilizing transformation, while the metabolomics dataset was log2-transformed after replacing zero values with half of the minimum non-null value in the dataset. NIPALS-PCA was used to impute missing values in the genomics and metabolomics datasets. In order to reduce the size of the genomics and transcriptomics datasets, a sparse PLS-DA analysis was performed on each dataset to select features that best separate the control and infected animals (Table S1, available as supplementary data at *Bioinformatics* online). For each dataset, the number of features to retain was set to 1000. An alternative version of this pre-filtered multi-omics dataset was created by retaining instead retaining the 1000 features with the highest median absolute deviation scores from each of these two datasets.

### 3.2 Data exploration

**Figure 2:**
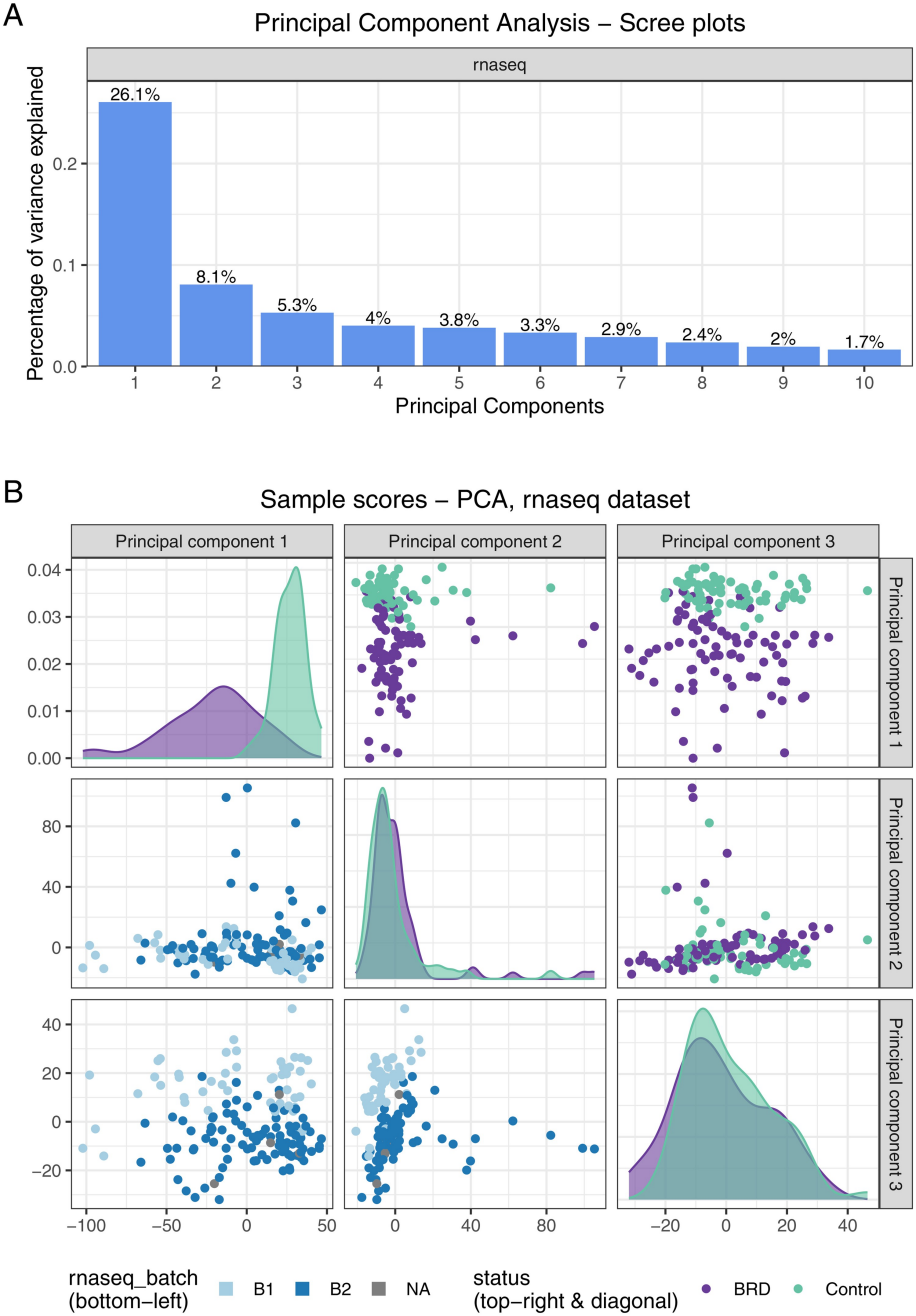
Results of a Principal Component Analysis on the beef cattle transcriptomics dataset. (A) Percentage of variance explained by each of the first 10 principal components. (B) Samples score for the first three principal components. In the bottom left plots, the colour of the points denotes the batch in which the samples were measured during the RNAseq analysis.

In the input datasets, some samples were only present in a subset of the omics datasets. An Upset plot generated with moiraine shows that 135 samples are common across all three omics datasets (Fig. S2, available as supplementary data at *Bioinformatics* online). The results of the PCA performed on each transformed but not filtered dataset allow us to quickly assess the main trends in each omics dataset. They notably highlight the presence of a batch effect in the transcriptomics dataset, which could influence the results of the analysis (Fig. 2). This was not explicitly checked in the original study, although the transformation applied to read counts was intended to reduce its impact.

### 3.3 Multi-omics integration results

The multi-omics dataset was analysed using four methods supported in moiraine: sPLS, sO2PLS, MOFA, and DIABLO. As sPLS and sO2PLS can integrate only two datasets, they were applied to the transcriptomics and metabolomics datasets. For DIABLO, which is a supervised method, the disease status of the animals (control or BRD-infected) was used as sample grouping. For MOFA, no groups were used. For all methods, only samples present across all three omics layers were used. The details of parameters used and parameter-selection steps are presented in Supplementary Material, available as supplementary data at *Bioinformatics* online.

**Figure 3:**
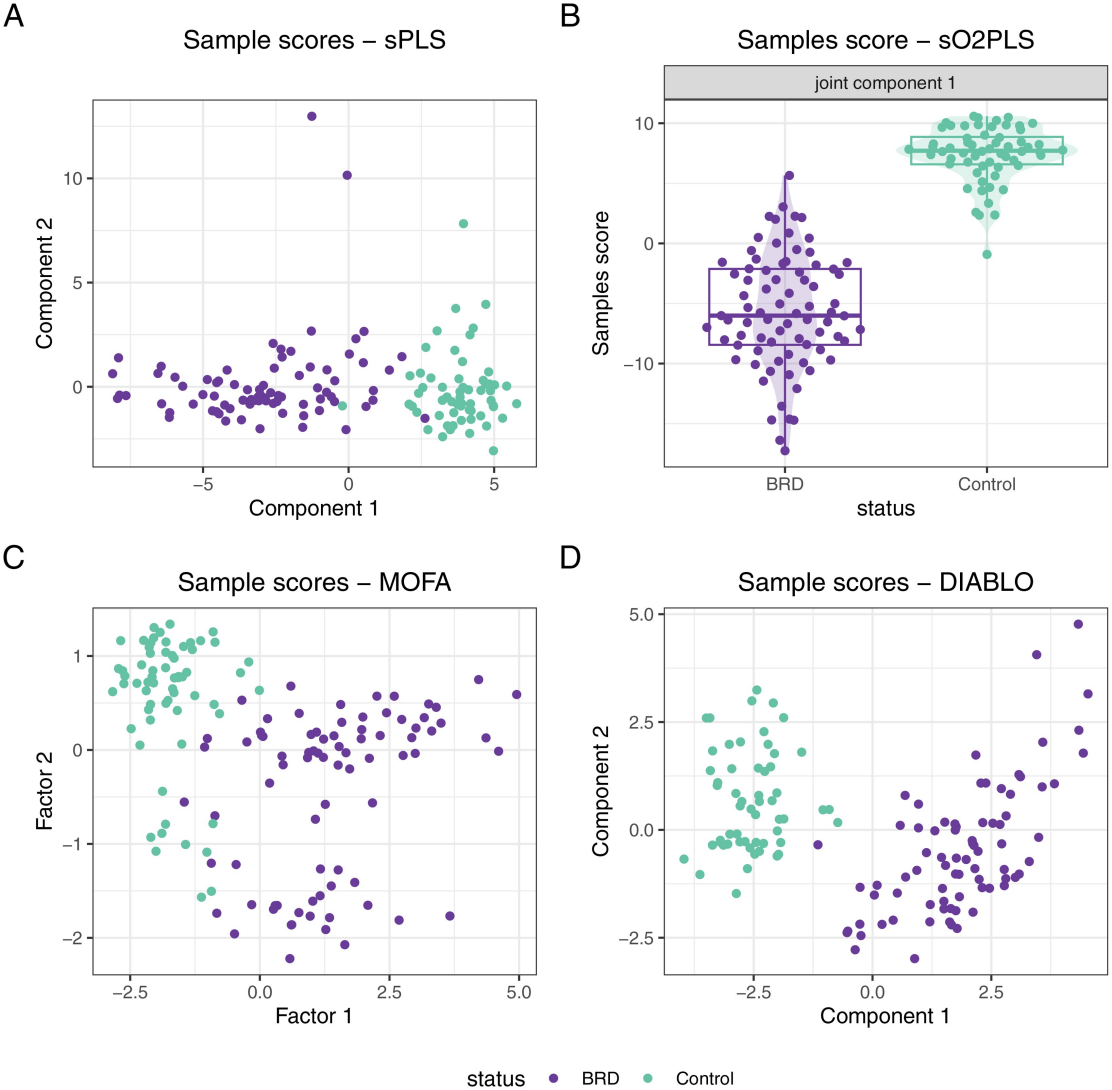
Sample scores for the first one or two latent dimensions constructed by (A) sPLS, (B) sO2PLS (only the joint component is shown, and sample scores are plotted against the animal disease status), (C) MOFA and (D) DIABLO. Samples are coloured according to the animal disease status, i.e. control or infected.

The tuning process led to constructing two latent dimensions with sPLS, seven with sO2PLS (one joint, one transcriptomics-specific, and five metabolomics-specific components), 15 with MOFA, and four with DIABLO (Tables S2–S11, available as supplementary data at *Bioinformatics* online). Across all methods, the first one or two latent dimensions separate the infected samples from the controls (Fig. 3). While this is expected in the case of DIABLO, as it is a supervised approach which maximizes the separation between the sample groups, the fact that it is also the case with the unsupervised approaches means that there is a strong biological signal in the datasets that differentiates the infected animals from the controls.

**Figure 4:**
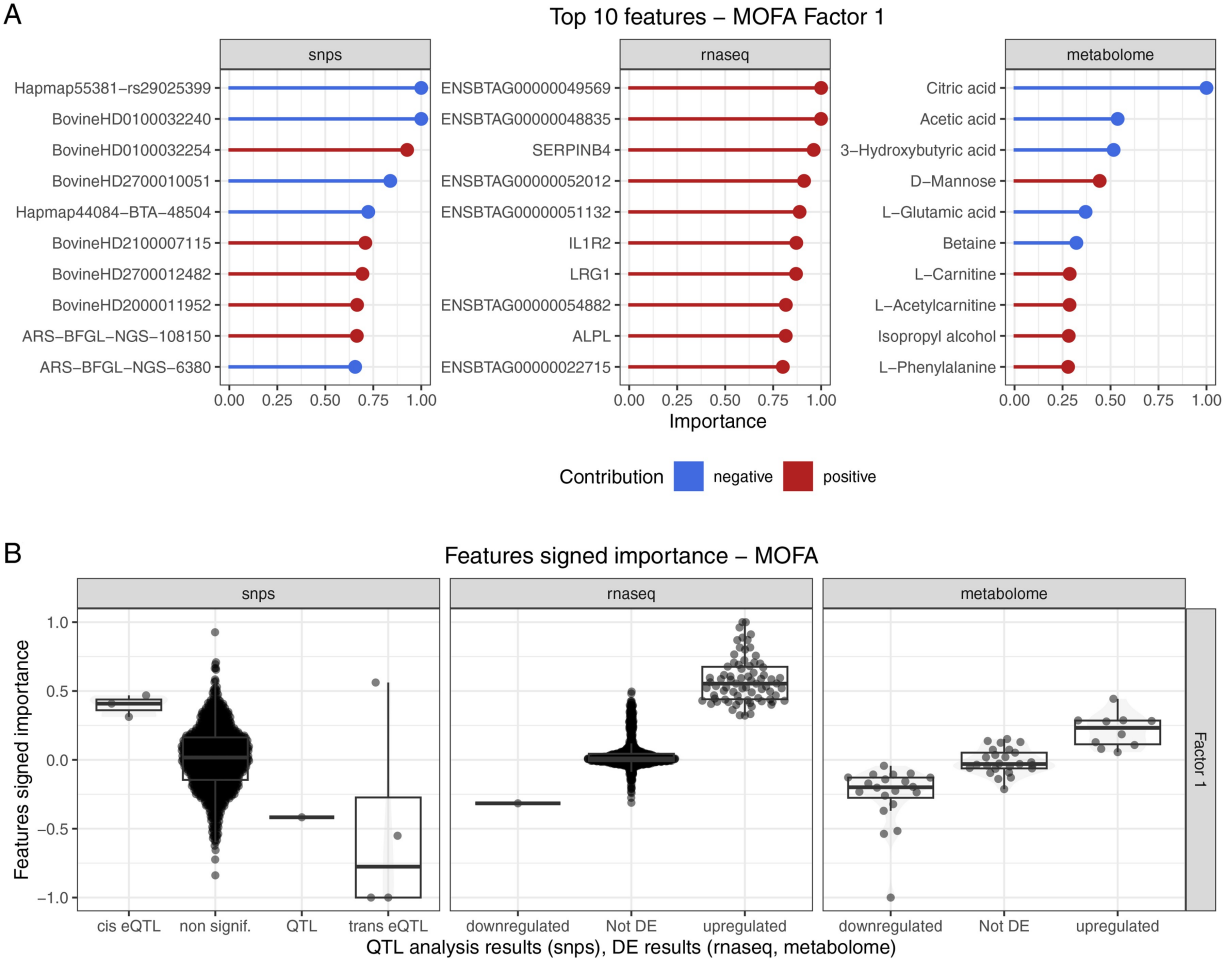
(A) Top 10 features from each omics dataset with the highest importance scores for MOFA factor 1. (B) Comparison between the feature importance scores for MOFA factor 1 and the QTL analysis results, transcriptomics and metabolomics differential expression results from Li *et al*. (2022). Note that only features that were retained after prefiltering are shown.

Taking MOFA as an example, the top contributing features for the first latent dimension (Factor 1—which separates the control and infected animals—are shown in Fig. 4A. These results can be compared with the results obtained in Li *et al.* (2022), which are stored in the features metadata of each omics dataset. We can see that the integration results are mostly in agreement with the single-omics results (Fig. 4B). In particular, two of the SNPs detected as trans-eQTL were assigned the highest importance score for MOFA factor 1, and all SNPs found as QTLs or eQTLs have importance scores above 0.3. However, some other SNPs not highlighted in the original study were given high importance scores. Similarly, transcripts and metabolites that were found to be differentially expressed show on average higher importance scores than non-differentially expressed features, even though some of these non-significantly DE features are assigned high importance scores. Among the latter might be features whose association with BRD could not be detected when analysing the omics datasets separately but which exhibit coordinated changes with features from other omics datasets. A gene set enrichment analysis was performed on the MOFA results, using the transcripts importance score for Factor 1 as weights (Table 2). Even though the enrichment *P*-values are not significant after correction for multiple testing, the results indicate that factor 1 prioritizes transcripts associated with keratinization, filament activity, and inflammatory response. Interestingly, keratinization has been linked to BRC in a previous study (Scott *et al.* 2021).

**Table 2:** Gene Ontology enrichment results for MOFA factor 1.^a^

GO term	GO term name	Statistics	*P*-value	Adjusted *P*-value	Number of genes in GO term
GO: 0031424	Keratinization	2.71	.00509	.786	20
GO: 0045109	Intermediate filament organization	2.44	.00938	.786	25
GO: 0010951	Negative regulation of endopeptidase activity	2.15	.0207	.786	25
GO: 0006954	Inflammatory response	1.86	.0318	.786	165
GO: 0050727	Regulation of inflammatory response	1.75	.0421	.786	56

a Only GO terms with a non-adjusted *P*-value below .05 are shown.

### 3.4 Comparing the integration results

**Figure 5:**
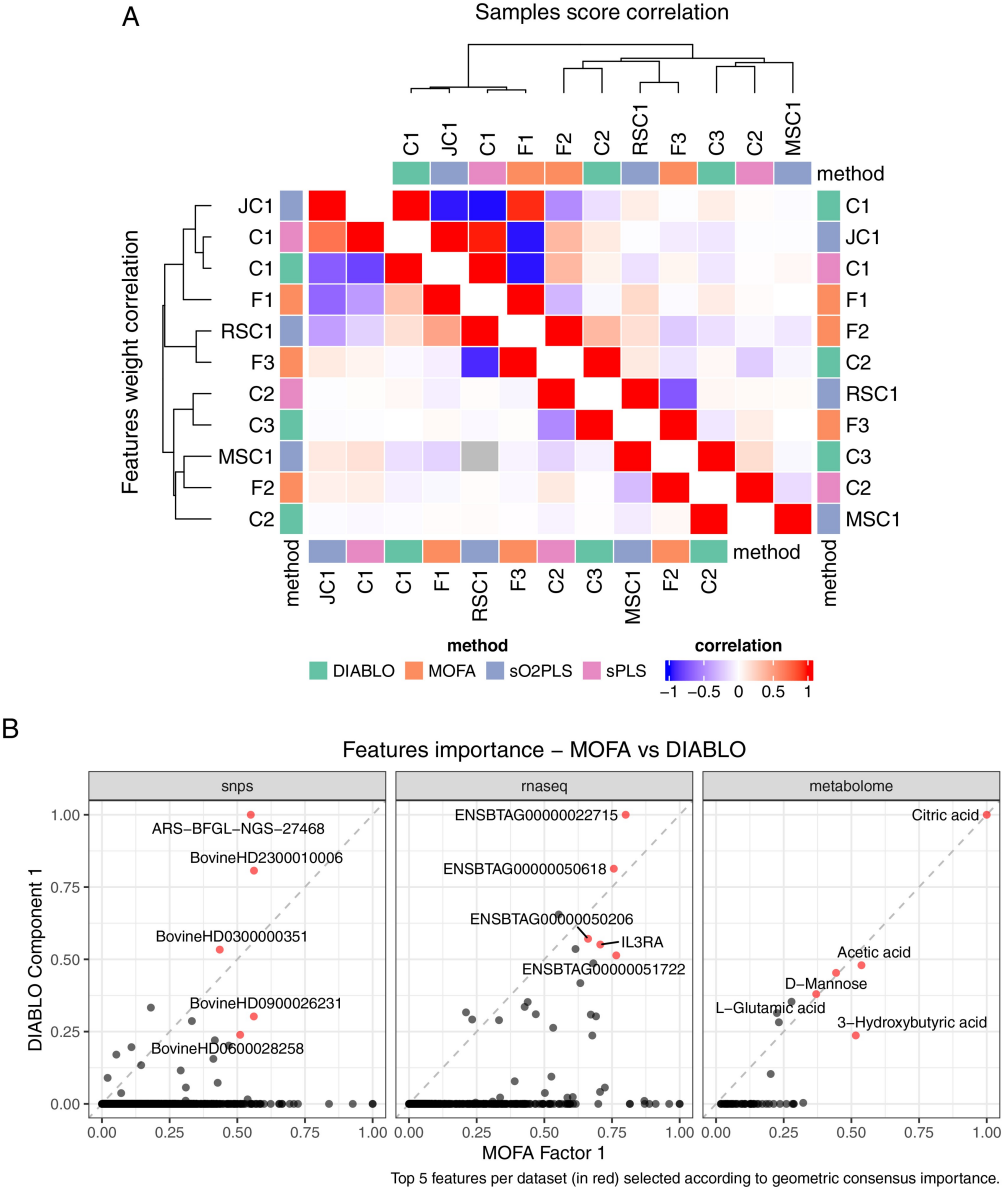
(A) Correlation heatmap between the feature weight (bottom left) or sample score (top right) of the first three latent components constructed with the different integration methods. The latent component labels are abbreviated as follows: C: component, JC: joint component, RSC: RNAseq-specific component, MSC: metabolome-specific component, F: factor. (B) Comparison between the feature importance score for the first latent component constructed with MOFA and DIABLO. The feature’s consensus importance score is computed as the geometric mean of their importance score across the two methods.

The correlation between the first three latent dimensions constructed with each method, both in terms of sample scores and feature weights, is shown in Fig. 5A. The first latent dimensions capture a similar trend across the samples, as can be seen with high correlations between their sample scores. However, their ranking of the features driving these trends are not in complete agreement, which is reflected in the lower correlations between their feature weights. These lower correlations are due in part to the feature selection performed by the methods (except MOFA), which drives most of the feature weights to 0. The subsequent latent dimensions of the different methods are less related, as each method seems to detect different trends in the data. However, there are still similarities, such as MOFA factor 3, which overlaps with the transcriptomics-specific component 1 constructed by sO2PLS.

The first latent dimension constructed with MOFA and DIABLO mostly agree on the feature ranking for the metabolomics dataset, with citric acid, acetic acid, and D-mannose being given the highest scores (Fig. 5B). On the other hand, a number of SNPs and transcripts that are given a high score with MOFA are not selected with DIABLO. This can be explained by the fact that DIABLO seeks to select features that are most associated with sample groups (here control versus infected), whereas MOFA tries to capture variation in the data. As there is more structure in both the genomics and transcriptomics datasets, reflecting notably the genomic composition of the animals, this might be captured in MOFA results more than in the DIABLO results.

A similar comparison can easily be repeated between the MOFA results obtained with the two different feature preselection approaches (Fig. S3, available as supplementary data at *Bioinformatics* online). Although the first two factors constructed are very similar regardless of the preselection method used, the subsequent factors differ strongly. This highlights the importance of choosing an appropriate preprocessing approach for multi-omics integration, as it strongly impacts the integration results.

### 3.5 Computational efficiency

The entire analysis pipeline ran in just under 2.2 hours on a Red Hat Enterprise Linux 9.5 system with four CPUs and 16.4 GB of memory. The most computationally intensive steps, both in terms of running time and memory usage, were the cross-validation runs for SNPs and genes prefiltering with sPLS-DA, as well as the cross-validation runs of DIABLO and sPLS (Fig. S4, available as supplementary data at *Bioinformatics* online). As prefiltering is applied to potentially very large datasets, it makes sense that it is one of the most resource-intensive steps of the pipeline. Here, the supervised prefiltering of the genomics and transcriptomics datasets—which have a similar number of features—took between 15 and 20 minutes per dataset. Additional testing of this prefiltering step on the larger version of the genomics dataset provides an indication of the scaling in time and memory usage when the number of features increases; namely, we observed a 4.2 times increase in running time and a 6.3 times increase in memory usage when the number of features was increased from 23 036 to 85 630 (Fig. S5, available as supplementary data at *Bioinformatics* online). This can be mitigated by first removing features with low variation using an unsupervised approach prior to performing supervised prefiltering or by reducing the maximum number of components to construct during the prefiltering with sPLS-DA. It must be noted that feature prefiltering is critical for the overall computational requirements of the pipeline, as reducing the size of the omics datasets will ensure a shorter running time and smaller memory requirement for all subsequent steps. In addition, prefiltering has been shown to improve the integration results by reducing the noise present in the data, which also reduces the risk of overfitting, and by reducing the imbalance between datasets, as the largest omics datasets would otherwise dominate the integration results (Sathyamoorthi *et al.* 2025, Subramanian *et al.* 2020, Rohart *et al.* 2017, Perez-Riverol *et al.* 2017). Therefore, it is strongly recommended to reduce the size of each omics dataset prior to integration.

**Figure 6:**
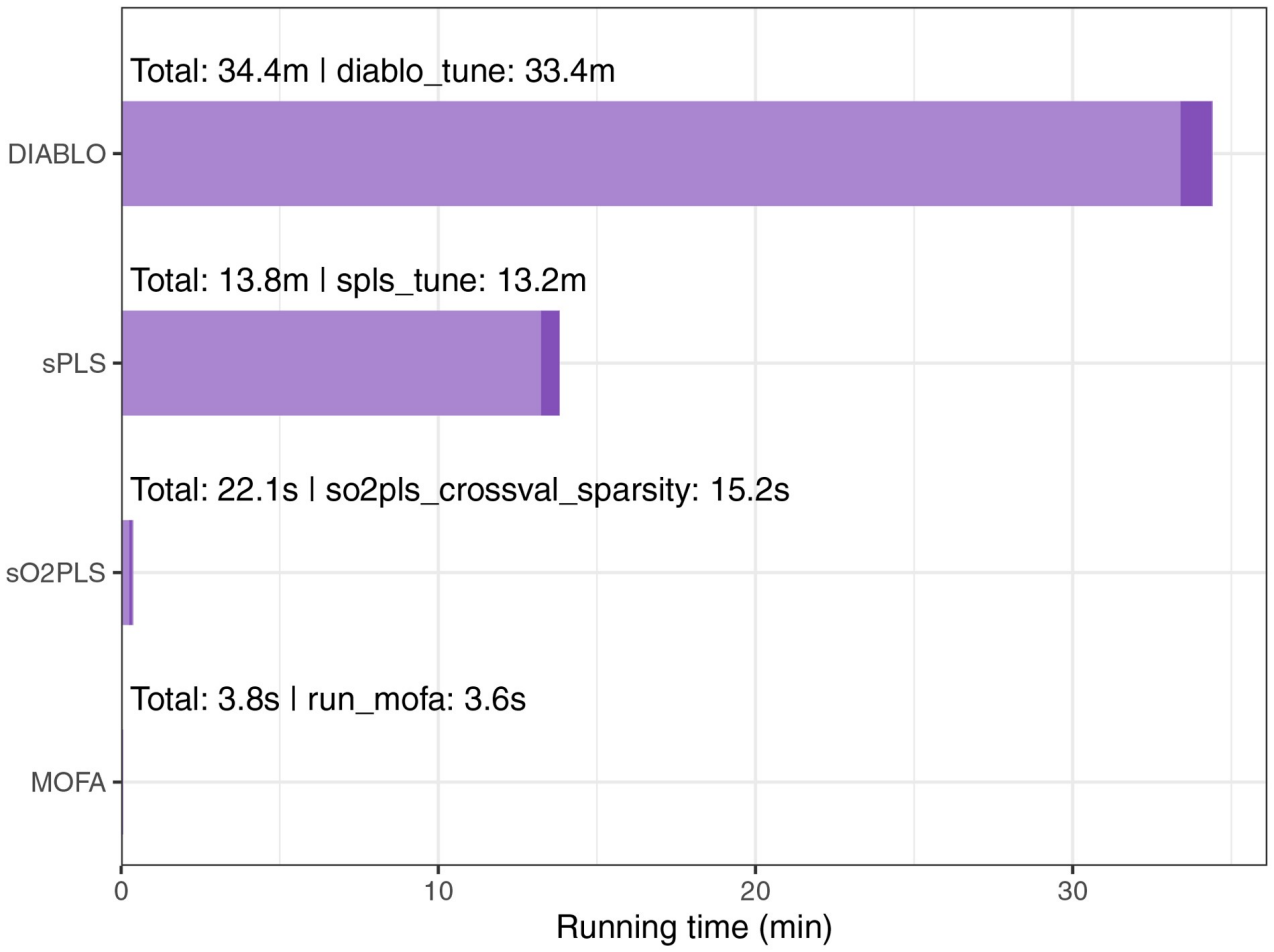
Running time of the different steps involved in the integration of the example multi-omics dataset via different tools (sPLS, sO2PLS, MOFA, DIABLO). The total running time for each tool is indicated, as well as the function with the longest running time for each tool (shown in a lighter shade).

Among the four integration tools tested, DIABLO was the slowest, with a total running time of around 34 minutes, including 33 minutes spent on cross-validation to select the optimal number of features to retain (Fig. 6). sPLS followed a similar pattern, with most of the running time (13.2 of 13.8 minutes) spent on this cross-validation step. This is, of course, dependent on the number of combinations of values to test; for large datasets or with many omics layers, the cross-validation running time can be reduced by testing a smaller number of values. In contrast, both sO2PLS and MOFA ran in under 1 minute. For sO2PLs, the longest step was also the cross-validation run to assess the optimal number of features to retain. Interestingly, both the slowest and fastest tools (DIABLO and MOFA) integrated all three omics datasets, while sPLS and sO2PLS can only integrate two omics datasets. To assess how an increase in the number of omics datasets to integrate affects running time, we applied both DIABLO and MOFA to a second multi-omics dataset, comprising eight different omics layers. For DIABLO, we reduced the cross-validation grid search to only two values per omics dataset, since two of these omics layers contain <50 features. The running time of DIABLO increased to 1.6 hour, again mostly due to the cross-validation step to select the number of features to retain; the running time of MOFA increased but remained under 1 minute (Fig. S6, available as supplementary data at *Bioinformatics* online).

## 4 Discussion

We present moiraine, an R package for constructing reproducible multi-omics integration analysis pipelines. moiraine enables users to import omics datasets and associated features and samples metadata, without any restriction on the type or number of datasets that can be included. These datasets can then be inspected, transformed and pre-filtered; a number of options are made available, but custom steps can easily be implemented in the analysis pipeline. moiraine handles the conversion of the multi-omics dataset into whatever input format is required for one of the supported integration methods, namely sPLS, sO2PLS, DIABLO, MOFA or MEFISTO, allowing the user to focus on data integration rather than data wrangling. It facilitates the application of these integration methods, providing helpful default settings, but the user retains full control over the parameters used.

The integration results can then be converted into a standardized format for dimension reduction output, which allows users to generate consistent visualizations regardless of the method used. These visualizations can be enriched with information extracted from the samples or features metadata, which facilitates their interpretation. In addition, the integration results can be evaluated against information about the samples (e.g. to assess the separation of different sample groups) or features (e.g. to compare with results from previous analyses) or against prior knowledge about the features through feature set enrichment. Importantly, moiraine enables the comparison of the different integration methods by assessing the overlap between the latent dimensions constructed, both in terms of the trends uncovered across samples and the ranking of the omics features. Furthermore, a consensus importance score can be computed to aggregate the results of different integration methods in order to obtain a robust prioritization of the omics features.

The generated analysis pipelines are modular and fully customizable by the user who has access to all the intermediate results and ensure the reproducibility of the analysis. During the construction of these pipelines, users are guided by the extensive documentation available online.

There are several avenues for further development of the moiraine package. First, while moiraine currently accommodates five widely used integration methods, support for additional tools will be implemented. Second, data import from more specialized formats will be enabled; the ability to read data directly from local or public databases could be envisioned in the future. Other functionalities for interpreting and evaluating the integration results can also be offered. In addition, more examples of the use of moiraine for specific scenarios will be made available to provide more guidance for new users. Finally, results from the benchmarking of computational requirements of the different integration methods highlighted areas of potential improvement. In particular, optimization of the most time-consuming step for the mixOmics methods (i.e. sPLS and DIABLO), namely the sparsity cross-validation step, would be beneficial in reducing the overall running time of the pipeline.

With moiraine, we hope to encourage users to test different integration methods on their own data and to compare the results in order to ensure the robustness of their findings.

## Supplementary Material

btag070_Supplementary_Data

## Data Availability

The moiraine R package is publicly available at https://github.com/Plant-Food-Research-Open/moiraine; an archival snapshot of the package is available on Zenodo at https://doi.org/10.5281/zenodo.17172718. The code used to reproduce all figures and results (including how to obtain the datasets used as examples) is available on GitHub at https://github.com/Plant-Food-Research-Open/moiraine_publication_code and as a supplementary file.
