# Supplementary material for: moiraine: an R package to construct reproducible pipelines for the application and comparison of multi-omics integration methods
Source: Bioinformatics. 2026 Feb 15;42(3):btag070. doi: 10.1093/bioinformatics/btag070 (PMC12960915; doi:10.1093/bioinformatics/btag070)
Supplement: btag070_Supplementary_Data [file btag070_supplementary_data.zip › supplementary_material_figures.pdf]

moiraine: an R package to construct reproducible pipelines for the application and comparison of multi-omics integration tools

Supplementary File 1 - Supplementary Material and Figures

O. Angelin-Bonnet, L. Guo, Roy Storey, S. Thomson

Description of supported multi-omics integration methods

sPLS

Sparse Partial Least Squares (sPLS) (Cao et al. 2008) is a method for the unsupervised integration of two omics datasets, implemented in the mixOmics package. Given two or more omics datasets for which measurements are taken on the same samples, sPLS aims at investigating the shared variation between the datasets through dimension reduction. It constructs latent components, which are linear combination of the omics features, such that the covariance between each omics dataset component is maximised. These latent components are constructed iteratively, and different matrix deflation modes can be used after the construction of each component. The selected mode defines the role that the two datasets play in the analysis, i.e. whether there is a predictor and a response dataset, or whether the two datasets play symmetrical roles. In addition, L1-regularisation is applied to features weight for each latent component in order to perform feature selection.

With moiraine, an input object for sPLS is constructed from the MultiDataSet object, with the user selecting the two datasets to be integrated, as well as the mode to be used for matrix deflation. Optionally, one or more covariates from the samples metadata can be selected to enable the multilevel option from the method, which handles repeated measurements. Since sPLS only accepts datasets with the same samples, the omics datasets will be restricted to their common samples during the construction of the sPLS input. Cross-validation is used to select the optimal number of latent components to construct, and then the number of features to retain from the omics datasets for each latent component. The selected values are used for the sPLS run.

sO2PLS

Sparse Orthogonal two-way Partial Least Squares (sO2PLS) (Bouhaddani et al. 2018) is a method for the unsupervised integration of two omics datasets, implemented in the OmicsPLS package. Developed as an extension of the sPLS method, it aims at decomposing each of the two datasets into a joint part, which reflects variation shared by the datasets, an orthogonal or specific part, which represents variation unique to each dataset, and a residual part. To this end, sets of joint and specific latent components are constructed for each dataset; these components are constructed as a linear combination of the features from the corresponding dataset. The joint latent components are constructed by maximising the covariance between the projection of each dataset onto their respective joint part, and feature selection is performed by applying L1-regularisation on the feature joint loadings (i.e. their contribution to the joint components). No sparsity constraint is used on the feature loadings for the specific components. Joint and specific components are iteratively updated, with the datasets being corrected by removing the specific part before re-calculating the joint components. While the number of joint components is the same for the two datasets, each dataset can have a different number of specific components, including no specific component at all.

With moiraine, the sO2PLS input is constructed from the MultiDataSet object, with the user selecting the two datasets to be integrated. Cross-validation is used to assess the optimal number of joint and dataset-specific components to construct. The authors of the package recommend a two-step approach for this: first, an adjusted cross-validation method is used to reduce the search space, then a traditional cross-validation approach is used to refine the results. Lastly, cross-validation is also used to select the optimal number of features to retain from each dataset to construct the joint components.

The OmicsPLS package currently offers very limited functionalities to visualise the sO2PLS results. In moiraine, we implemented a number of options to visualise a fitted sO2PLS model, including diagnostic plots for the different cross-validation steps, a summary of the variance explained by the fitted model, and a graph of the relationship between the datasets' joint components.

DIABLO

The Data Integration Analysis for Biomarker discovery using Latent Components (DIABLO) method (Singh et al. 2019) is a multivariate approach to perform supervised data integration, implemented in the mixOmics package. Given two or more omics datasets for which measurements are taken on the same samples, and a list of sample affiliation to an outcome group, DIABLO aims at selecting correlated features across the datasets that best discriminate the different sample groups. DIABLO works by iteratively constructing linear combinations of the features, called latent components, that maximise the correlation between the datasets and with the categorical outcome. In order to perform feature selection, the latent components are subjected to L1-regularisation, i.e. the number of features included in the linear combination

is constrained by the user. Moreover, the optimisation problem is weighted to allow users to control the balance between the two objectives of 1) maximising the correlation between omics datasets and 2) discriminating between the outcome groups of interest.

In the moiraine package, the MultiDataSet object is used to generate an input for the DIABLO algorithm. Information about the samples outcome groups are extracted from the samples metadata associated with the omics dataset, based on user input. As DIABLO only accepts datasets in which the same samples are present, the omics datasets are restricted to their common samples only. By default, all omics datasets are used, but the user can instead choose a subset of them for analysis.

One of the required input parameters for the DIABLO algorithm is the design matrix, which is used to balance the two objectives of the method (maximising the correlation between the datasets and maximising the discrimination between the sample groups). In the pipeline, the user can either select a predefined design matrix (as proposed by the authors of the mixOmics package), or estimate the design matrix from the datasets. This is achieved through pairwise comparison of the omics datasets via projection to latent structures (PLS), also implemented in the mixOmics package. For each pair of omics datasets, the PLS run is used to estimate the correlation between the datasets. The design matrix is then populated by applying a threshold on the estimated correlations.

The optimal number of latent components to construct, as well as the optimal number of features to retain from the omics datasets for each latent component, can be estimated by cross-validation. For both aspects, moiraine implements functions to visualise the cross-validation results, and to extract the optimal value from the results. These optimal values are then used for the DIABLO run.

MOFA

Multi-Omics Factor Analysis (MOFA) (Argelaguet et al. 2018) is a method for the unsupervised integration of two or more omics datasets, implemented in the MOFA2 package. It aims at uncovering the main axes of variation shared by all or a subset of the datasets, through the construction of a small number of latent factors. It can be assimilated to a generalisation of the PCA to multiple datasets. The latent factors are constructed as sparse linear combinations of the features. MOFA uses a Bayesian framework to decompose each dataset into the product of a latent factor matrix, which corresponds to the main axes of variations, and a matrix of feature weights, which indicate the extent to which the features contribute to the different latent factors. MOFA applies two levels of sparsity. The first level of sparsity is used to detect whether a given latent factor is active (i.e. explains variation) in each dataset. This is done to assess which sources of variations are shared across the datasets, or unique to some datasets. The second level of sparsity is applied to the features, to assess which features contribute to the different sources of variation. Within MOFA, the multi-group framework

enables the comparison of different groups of samples, in order to detect trends shared by the different groups.

With moiraine, a MultiDataSet object is used to generate the MOFA input data. The user can choose which datasets should be included in the analysis, and which column of the samples metadata should be used to inform sample grouping when using the multi-group framework. As MOFA doesn't require samples to be present in all omics datasets, the user can also decide whether to include all samples present in the MultiDataSet object or whether to restrict the analysis to only samples that are present in all omics datasets. In addition, all the tuning parameters that can be controlled by the user for the MOFA model, such as the likelihood function to use for each omics dataset or the maximum number of latent factors to construct, can be set during the construction of the MOFA input. The MOFA model is then fitted through the MOFA2 package.

MEFISTO

The MOFA2 package also offers an extension of MOFA, called MEFISTO (Velten et al. 2022), which explicitly accounts for a continuous structure amongst the samples (i.e. spatial or temporal relationship). MEFISTO works in the same way as MOFA to construct latent factors expressing variation trends in the datasets, but in addition assesses whether the constructed factors are related to or independent of the samples continuous structure. It can also use this information to infer information about unobserved locations or time-points.

In addition to the parameters that can be set for a MOFA model, MEFISTO requires as input one or two continuous covariates that describe the relationship between the samples: e.g. a time covariate, or coordinates for a 2D spatial structure. A Gaussian process is used as prior during the construction of the latent factors, and for each factor, a scale or smoothness parameter controls the extent to which the factor is related to the covariate(s).

In the moiraine package, the MEFISTO input is constructed from a MultiDataSet object in a similar way as for MOFA, but in addition the continuous covariates are extracted from the samples metadata as specified by the user. The MEFISTO model is then fitted through the MOFA2 package.

Beef cattle multi-omics dataset

Description of the original study

Li et al. (2022) investigated the molecular mechanisms of bovine respiratory disease (BRD) in beef cattle, using multi-omics data. Animals enrolled into four commercial beef cattle feedlots in Alberta, Canada in 2015 were used for this study. Animals were fed a specific diet during their stay in the feedlots. 80 animals were identified as BRD positive, and 63 healthy

individuals were selected from pens that had infected animals. Out of the 143 animals, 87 were heifers and 56 were steers. Blood samples were collected from each selected individual, and the following omics measurements were collected:

- genomics data (Illumina GGP Bovine 100K microarray SNP chip). Four samples were lost for this dataset;
- transcriptomics data (paired-end RNAseq data on total RNA). The samples were processed in two batches, with a different sequencing platform used for each batch (Hiseq 4000 and Novaseq 6000);
- metabolomics data (via NMR spectroscopy, using a 700 MHz Avance III spectrometer).

The genomics and metabolomics datasets were deposited in the borealis database (DOI: 10.5683/SP3/ZETWNY), and the transcriptomics dataset in the GEO database (accession GSE217317).

The genomics dataset was used to infer the genomic breed composition of the animals, using the ADMIXTURE software with ancestry value set to 3. Then, SNPs were called from the RNAseq data, and were combined with the genomics dataset for further analysis.

A genome-wide association study (GWAS) was run to identify SNPs significantly associated with disease status; the latter was adjusted for feedlot, days on feed and genomic breed composition. Two SNPs with a false discovery rate (FDR) < 0.05 were considered as significant quantitative trait loci (QTLs).

The transcriptomics data were used for a differential expression analysis, accounting for feedlot, genomic breed composition and sequencing batch. The group of healthy samples was used as the reference in the analysis; 101 genes with an FDR < 0.01, log2 fold-change > 2 and log-counts per million > 2 were considered as significantly differentially expressed (DE).

An expression-QTL (eQTL) study was performed on the DE genes to identify SNPs significantly associated with their expression, again correcting for feedlot, sequencing batch and genomic breed composition. 564 SNPs with an FDR < 0.05 were considered as significant eQTLs. The physical distance between an eQTL and the associated gene was used to classify the mode of action of the SNP: 420 SNPs located within 1Mbp of the gene transcription starting site were considered cis-eQTLs, those located further or on different chromosomes (144 SNPs) were instead classified as trans-eQTLs.

A differential concentration analysis was performed on the metabolomics data using two-samples t-test, and adjusting for feedlot and sex. The group of healthy samples was used as reference; 35 metabolites with an FDR < 0.05 were considered as DE.

Data preparation for multi-omics integration with moiraine

The following section details how the data made available by Li and co-authors was extracted and processed in preparation for analysis with moiraine. The full script for the data processing can be found in the moiraine GitHub repository.

Genomics dataset

The matrix of SNP dosage and table of SNP information were downloaded from the borealis database. The list of significant QTLs and eQTLs was extracted from the supplementary file made available on Figshare alongside the article and completed with information from Table 1 from Li et al. (2022). Note that some of the QTLs and eQTLs listed in the article and supplementary material were obtained from the RNAseq dataset, and therefore are not present in the version of the dataset used for the present tutorial. If a SNP was selected as both QTL and eQTL, only the results yielding the smallest FDR value were retained.

SNPs with more than 10% of missing values, a minor allele frequency lower than 5%, or located on a sex chromosome were removed from the analysis, to reproduce the preprocessing described in the original paper's methods; however filtering based on Hardy-Weinberg equilibrium was not performed. In addition, to keep the size of the dataset small (as this dataset is indented for demonstration only), 23,000 SNPs that were not found significant QTLs or eQTLs were randomly subsampled from the full set of non-significant SNPs. All SNPs detected as QTLs or eQTLs were also retained in the dataset; yielding a genomics dataset of 23,036 SNPs over 139 samples.

K-means clustering was used to group samples into 3 clusters, based on their genomic breed composition values.

Transcriptomics dataset

The matrix of RNAseq read counts, alongside information about the measured samples, was downloaded from the GEO database. Sample IDs were corrected when possible to match with the IDs found in the datasets from the borealis database, based on information about the animals. Genes with a read count of 0 for 90% or more samples were removed from the dataset. This resulted in a dataset of 20,335 genes over 143 samples.

The genome annotation file used for mapping the RNAseq reads (Bos taurus genome, version 110) was downloaded from the Ensembl database. A lighter version of the file was generated by only retaining information about the genes in the GFF3 file. In addition, the genes' GO annotation was obtained through the `biomaRt` package using the Bos taurus Ensembl genome annotation (version 110).

The differential expression analysis was re-created according to the manuscript's methods section, using the edgeR package. Due to difference in package version and missing information about two samples, the results are not completely identical, nevertheless, most of the DE genes overlapped with the ones reported in the manuscript.

Metabolomics dataset

The table of metabolites concentrations was downloaded from the borealis database, containing measurements for 55 metabolites over 139 samples. Information about the measured metabolites was found in the HMDB dataset (https://hmdb.ca/). The contents of the database were downloaded and parsed to extract relevant properties of the measured metabolites.

The differential expression analysis on the compounds was re-created according to the manuscript's methods section, using a two-sample t-test.

Resulting files

The following files were generated:

- `genomics_dataset.csv`: contains the genomic variants' dosage, with 23,036 genomic variants as rows and 139 samples as columns.
- `genomics_features_info.csv`: contains information about the genomic variants (chromosome, genomic position, etc, as well as the results of a GWAS analysis), with 23,036 genomic variants as rows.
- `transcriptomics_dataset.csv`: contains the genes' raw read count, with 20,335 genes as rows and 143 samples as columns.
- `bos_taurus_gene_model.gff3`: the genome annotation file used to map the transcriptomics reads to gene models.
- `transcriptomics_de_results.csv`: the results of a differential expression analysis run on the transcriptomics dataset to compare healthy and infected animals, with 20,335 genes as rows.
- `transcriptomics_go_annotation.csv`: contains the correspondence between genes and GO terms in a long format (one row per gene/GO term pair).
- `metabolomics_dataset.csv`: contains the area peak values, with 139 samples as rows, and 55 compounds as columns.
- `metabolomics_features_info.csv`: contains information about the 55 compounds in rows (such as mass, retention time, and formula and name if the compound has been identified) as well as the results of a differential expression analysis run on the metabolomics dataset to compare healthy and infected animals.

- `samples_info.csv`: information about the samples, with 143 samples as rows.

These files are available through the moiraine package; after installing the package, they can be retrieved in R via the command `system.file("extdata/genomics_dataset.csv", package = "moiraine")`. Alternatively, they can be downloaded from the moiraine GitHub repository at https://github.com/Plant-Food-Research-Open/moiraine/tree/main/inst/extdata.

Analysis of the beef cattle dataset

Importing the data

The first step of the pipeline is to read in the different omics datasets and associated metadata. For each dataset, we need to import:

- the dataset itself (i.e. the matrix of omics measurements);
- the features metadata, i.e. information about the features measured in the dataset;
- the samples metadata, i.e. information about the samples measured in the dataset.

Typically, omics datasets as well as associated metadata are stored in csv files, although moiraine can also extract genes or transcripts information from GTF/GFF file. Once imported into R, the datasets and associated metadata are combined in a MultiDataSet object from the MultiDataSet package (Hernandez-Ferrer et al. 2017) which will be used in the rest of the analysis pipeline:

```
Object of class 'MultiDataSet'
 . assayData: 3 elements
    . snps: 23036 features, 139 samples
    . rnaseq: 20335 features, 143 samples
    . metabolome: 55 features, 139 samples
 . featureData:
    . snps: 23036 rows, 13 cols (feature_id, ..., p_value)
    . rnaseq: 20335 rows, 15 cols (feature_id, ..., de_signif)
    . metabolome: 55 rows, 16 cols (feature_id, ..., de_signif)
 . rowRanges:
    . snps: YES
    . rnaseq: YES
    . metabolome: NO
 . phenoData:
    . snps: 139 samples, 10 cols (id, ..., geno_comp_3)
    . rnaseq: 143 samples, 10 cols (id, ..., geno_comp_3)
    . metabolome: 139 samples, 10 cols (id, ..., geno_comp_3)
```

Data transformation

The datasets are transformed as follows:

- the Variance Normalising Transformation (VST) implemented in the DESeq2 package is applied to the transcript read counts;

- zeros in the metabolomics datasets are replaced with a small value, calculated as half of the minimum non-null value in the dataset; the dataset is then log2-transformed.

No transformation is applied to the genomics dataset. A density plot of the datasets after transformation is shown in Figure 1.

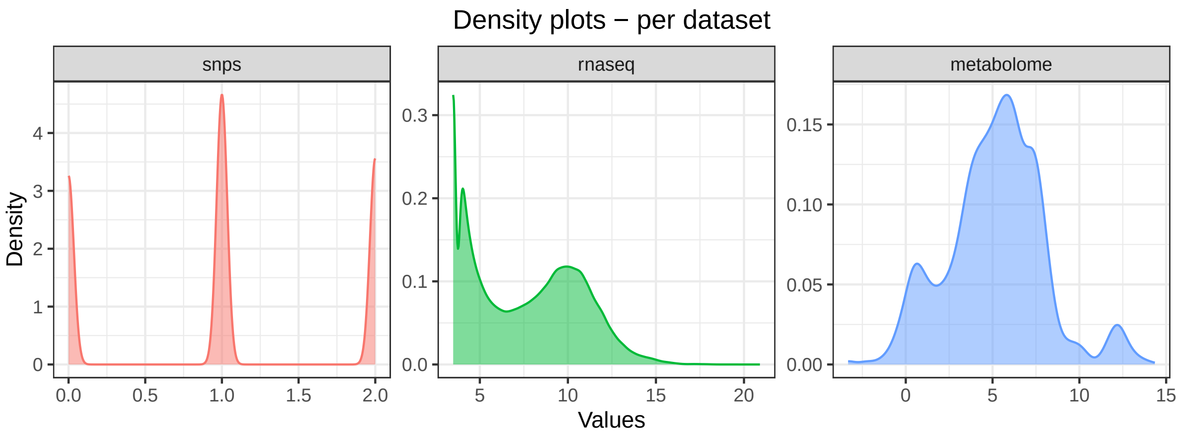

Figure 1: Density plot for each omics dataset after transformation of the transcriptomics and metabolomis datasets.

Missing values imputation

A PCA is applied to each transformed dataset separately. As the genomics and metabolomics datasets both contain missing values, a non-linear iterative partial least squares (NIPALS) method is used, which imputes missing values based on the results of the PCA. An overview of the PCA results for the genomics, transcriptomics and metabolomics dataset is shown in Figure 2, Figure 3 and Figure 4, respectively. A new MultiDataSet object is generated, containing the complete version of the omics datasets, i.e. in which missing values have been imputed.

A)

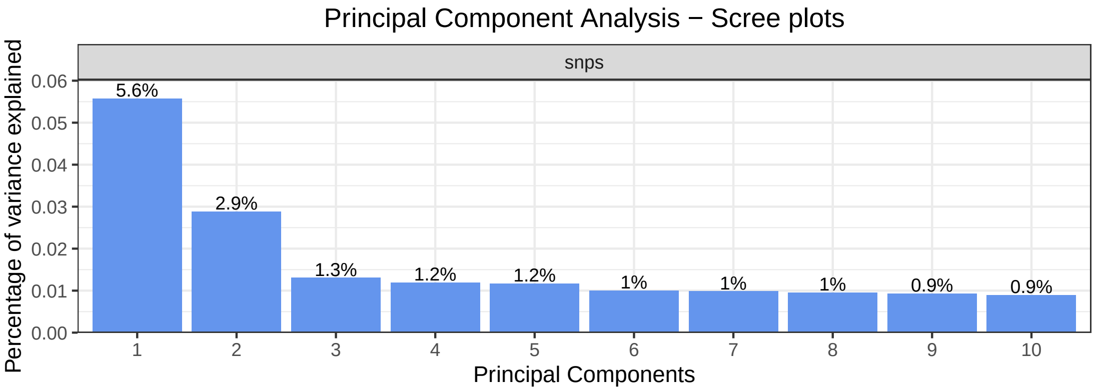

B)

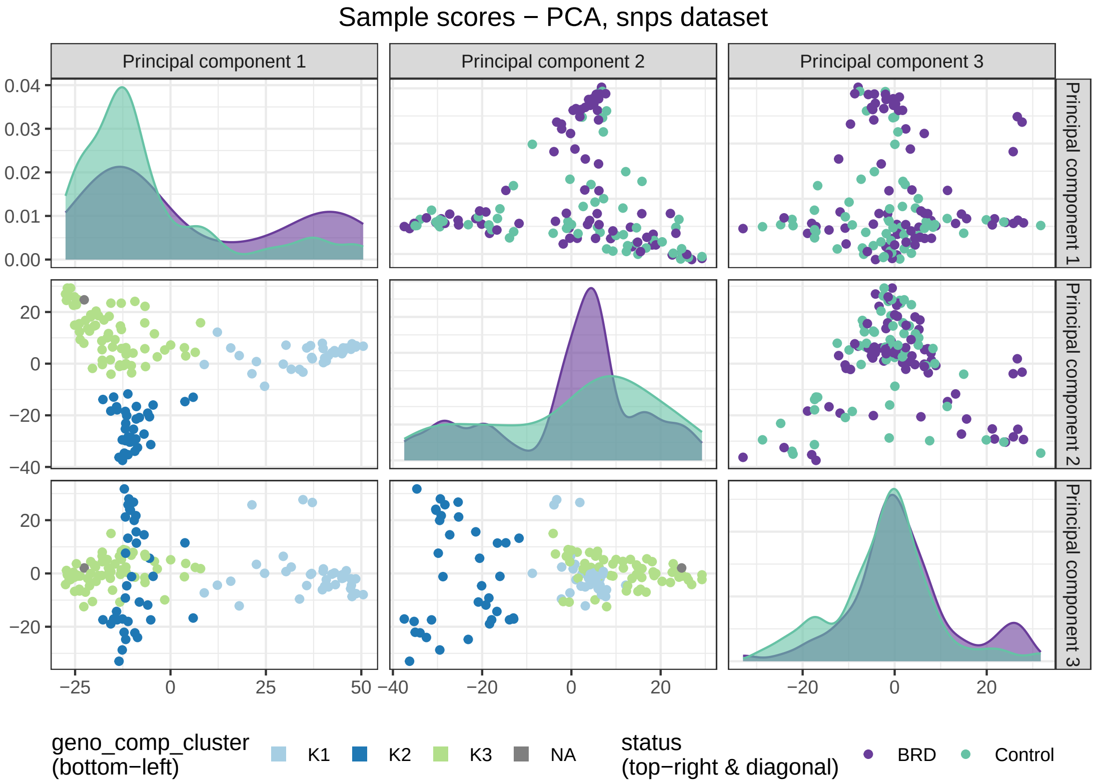

Figure 2: PCA results for the genomics dataset. A) Percentage of variance explained by the first ten principal components. B) Samples score for the first three principal components. In the bottom left plots, the colour of the points denote the cluster to which the samples were assigned based on their genomic composition. In the top right plots, the samples are instead coloured according to the disease status of the animal.

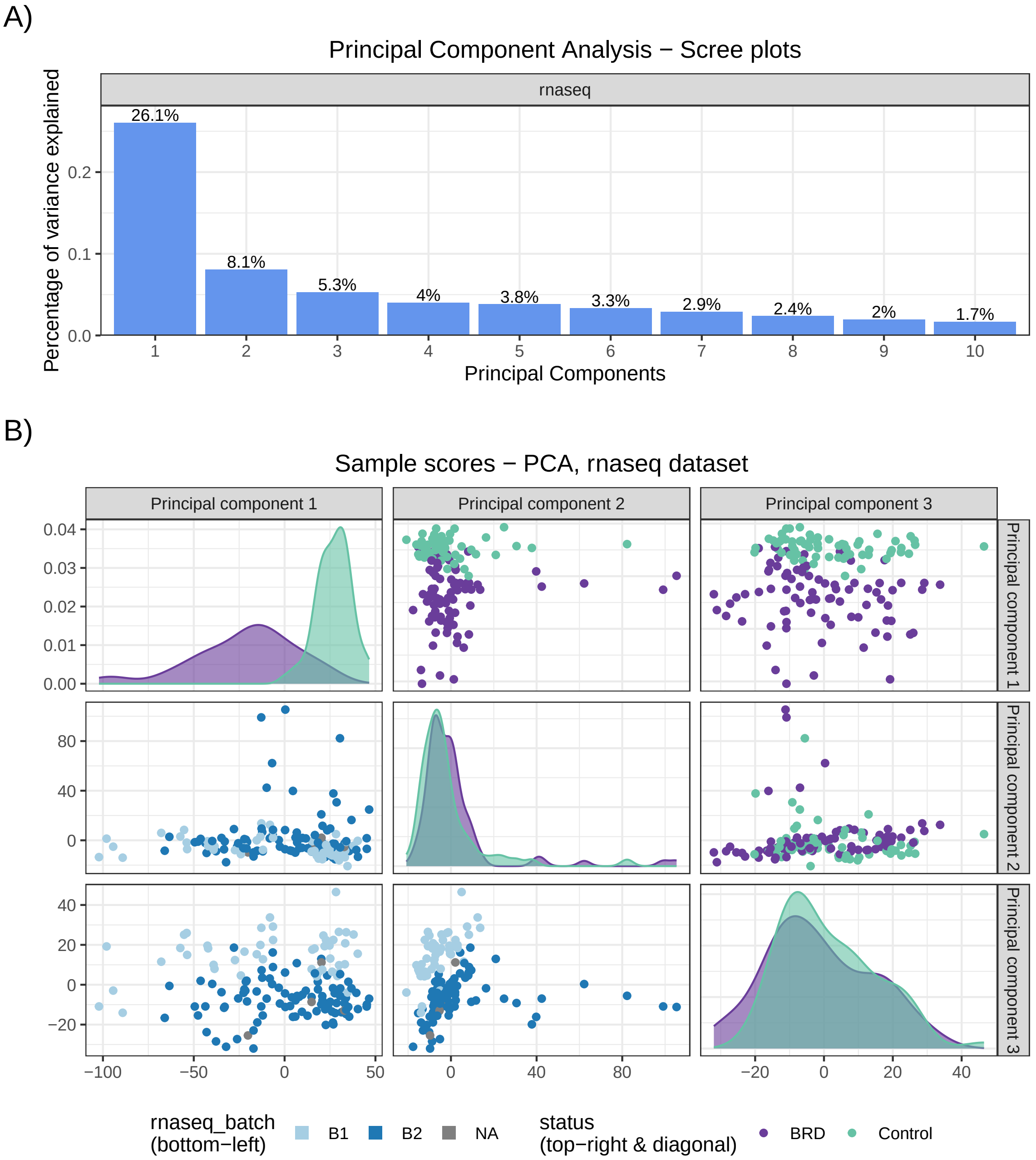

Figure 3: PCA results for the genomics dataset. A) Percentage of variance explained by the first ten principal components. B) Samples score for the first three principal components. In the bottom left plots, the colour of the points denote the batch in which the samples were measured during the RNAseq analysis. In the top right plots, the samples are instead coloured according to the disease status of the animal.

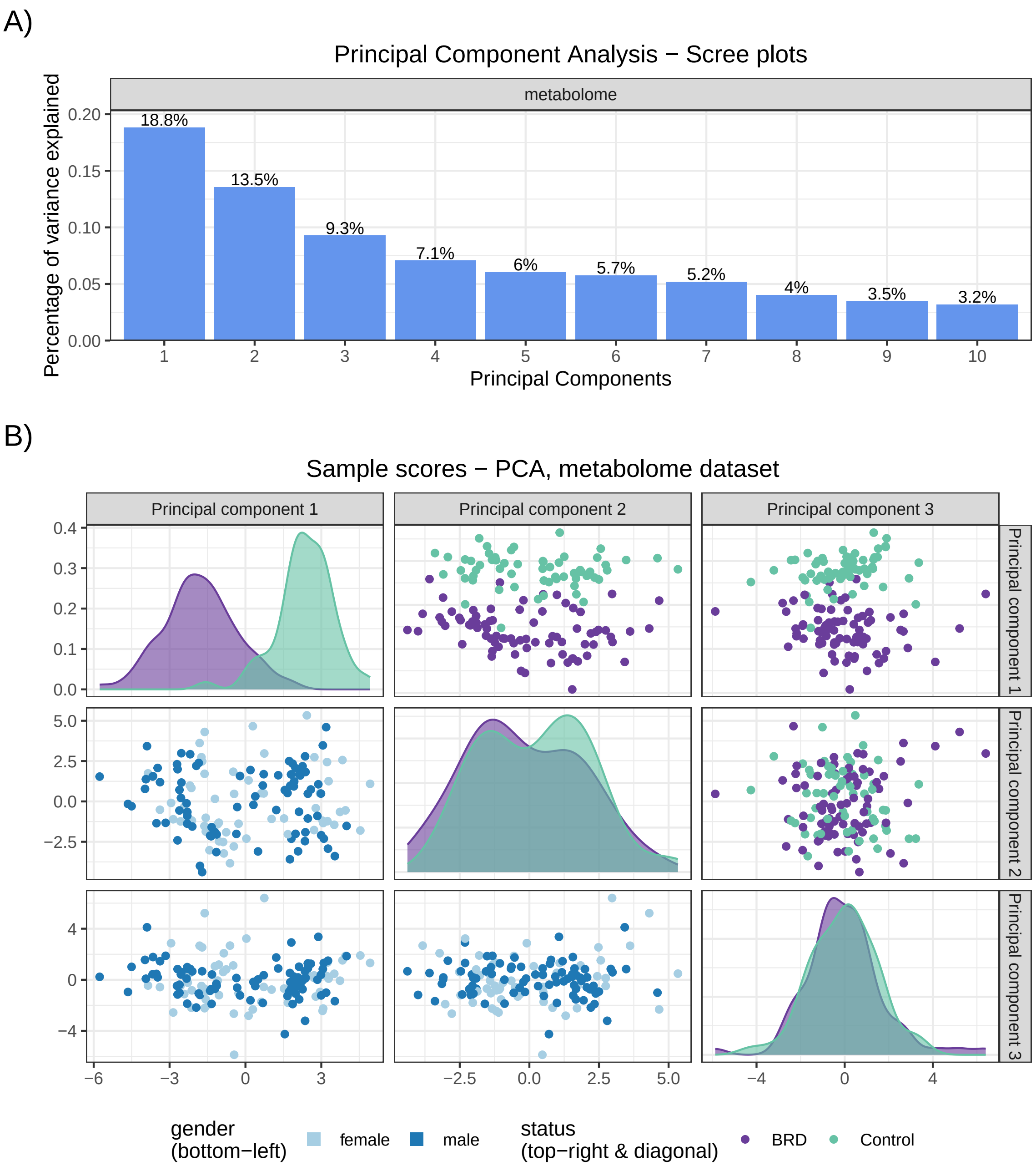

Figure 4: PCA results for the genomics dataset. A) Percentage of variance explained by the first ten principal components. B) Samples score for the first three principal components. In the bottom left plots, the colour of the points denote the gender of the animal. In the top right plots, the samples are instead coloured according to the disease status of the animal.

Features pre-filtering

In order to reduce the size of the datasets prior to integration, features pre-filtering is performed on the genomics and transcriptomics dataset. We use a supervised approach, aiming to retain features that are most associated with a sample outcome of interest–here, the disease status of the animals. This is done by applying a sparse Partial Least Squares Discriminant Analysis (sPLS-DA–implemented in mixOmics) on each dataset to be filtered, using disease status as the samples grouping to predict.

First, 5-fold cross-validation, repeated 50 times, is applied to select the appropriate number of latent components to construct. Values ranging from 1 to 5 are tested. The optimal value is selected using the centroids distance to assign samples to a group, and the balanced error rate (BER) to assess samples classification error. The cross-validation results lead to constructing one latent component for the genomics dataset, and five for the transcriptomics dataset Figure 5. A final sPLS-DA run is performed, using these values, by setting the number of features to retain to 1,000. In the case of the transcriptomics dataset, this number is split by moiraine between the five latent component, so that 200 features are set to be retained from each latent component. The set value is not necessarily the exact number of features retained, as some features can be selected for more than one latent component; consequently, the pre-filtered genomics and transcriptomics datasets contain 1,000 SNPs and 994 transcripts, respectively.

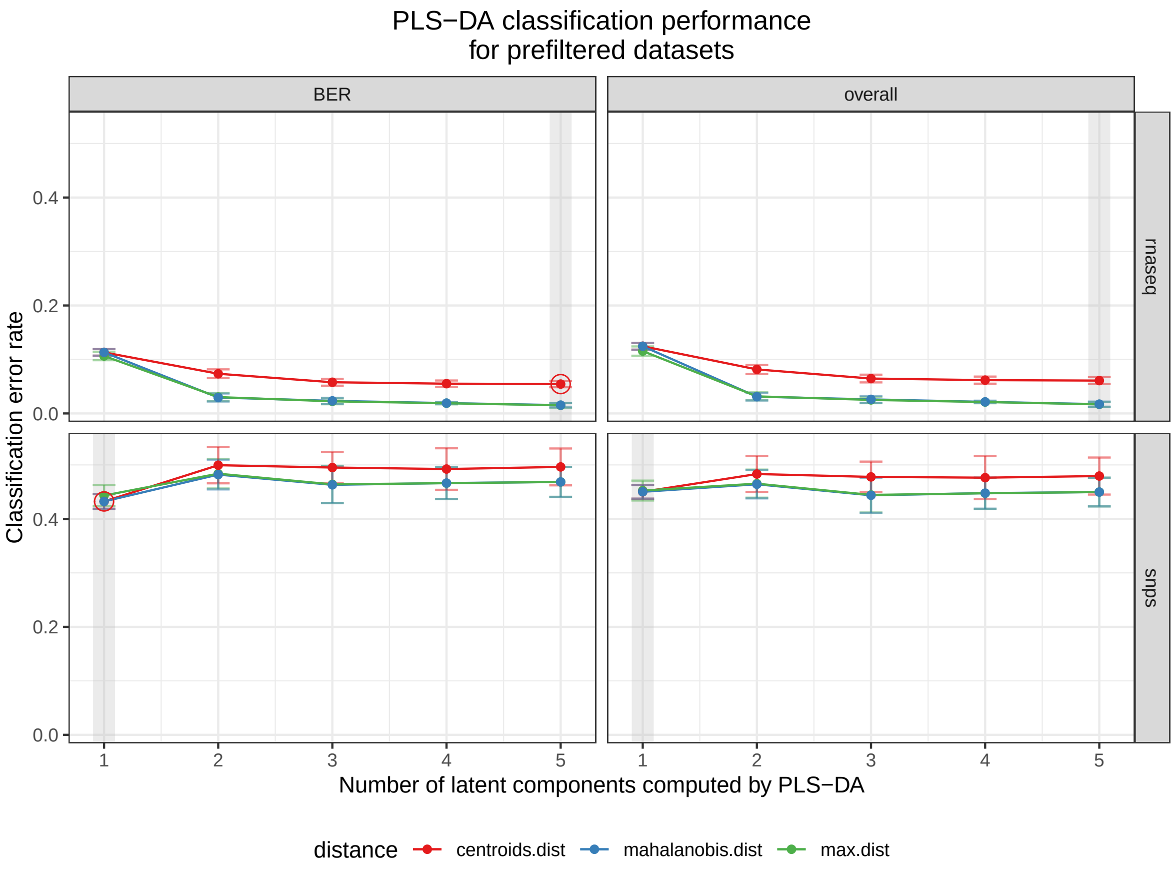

Figure 5: Cross-validation results for sPLS-DA run on the genomics (bottom plots) and transcriptomics (top plots) datasets. The left and right panels depict two different metrics that can be used to assess classification error, namely the balanced error rate and overall error rate.

In addition, we perform an alternative unsupervised pre-filtering, which aims to retain the most variable features in each dataset. Features with the highest Median Absolute Deviation (MAD) score are selected from the genomics and transcriptomics datasets. In both case, we set the number of features to retain to 1,000; ties are to be kept in the datasets. The pre-filtered genomics and transcriptomics datasets contain 12,618 SNPs and 1,000 transcripts, respectively.

Multi-omics integration with sPLS

The transformed and pre-filtered multi-omics dataset is used to generate an input object for sPLS; the transcriptomics and metabolomics datasets are selected for the analysis. The sPLS

mode is set to canonical, i.e. the two datasets are set to play a symmetrical role in the analysis. moiraine automatically removes any samples that are not present across all omics datasets.

Chossing the number of latent components

The optimal number of latent components to construct is selected via 10-fold cross-validation, repeated 10 times. Values from 1 to 4 are tested; the optimal value is selected using the Q^2_{total} criterion to quantify the predictive power of the model (Figure 6). As a result, the number of latent components to construct is set to 2.

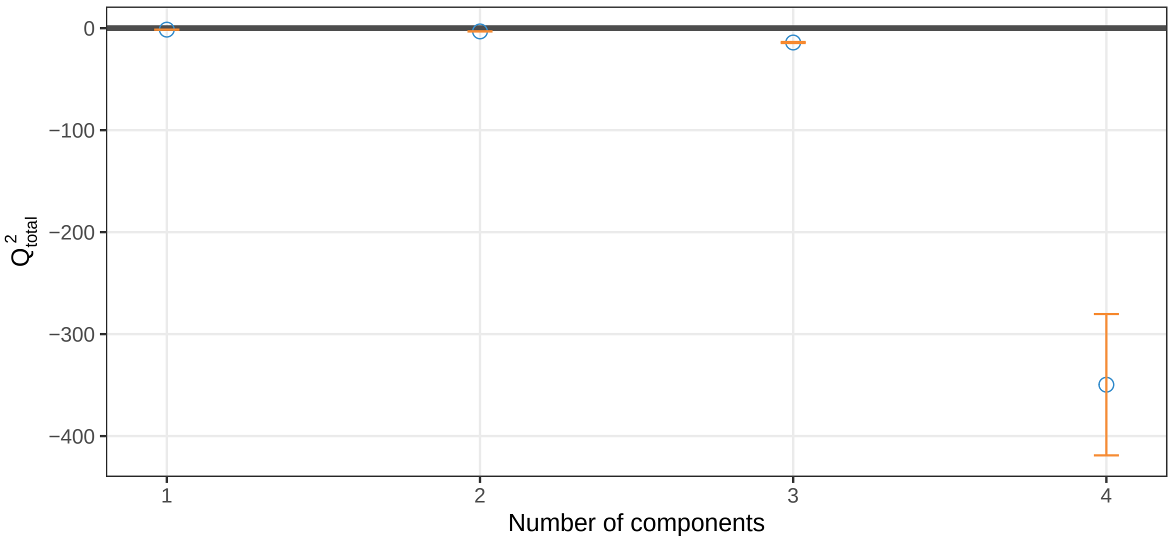

Figure 6: Cross-validation results for choosing the number of latent components to construct with sPLS.

Choosing the number of features to retain

The optimal number of features to retain from each omics dataset for each of the two latent components is selected via 10-fold cross-validation, repeated 5 times. Values from 10 to 100 by increment of 10 are tested; the optimal combination of values for each latent component is selected using the correlation between predicted and actual components as tuning metric (Figure 7). The values selected are shown in Table 3.

Table 1: Optimal number of features to retain from each omics dataset for each sPLS latent component, selected via cross-validation.

Dataset	Component 1	Component 2	Total
rnaseq	40	10	50
metabolome	20	50	70

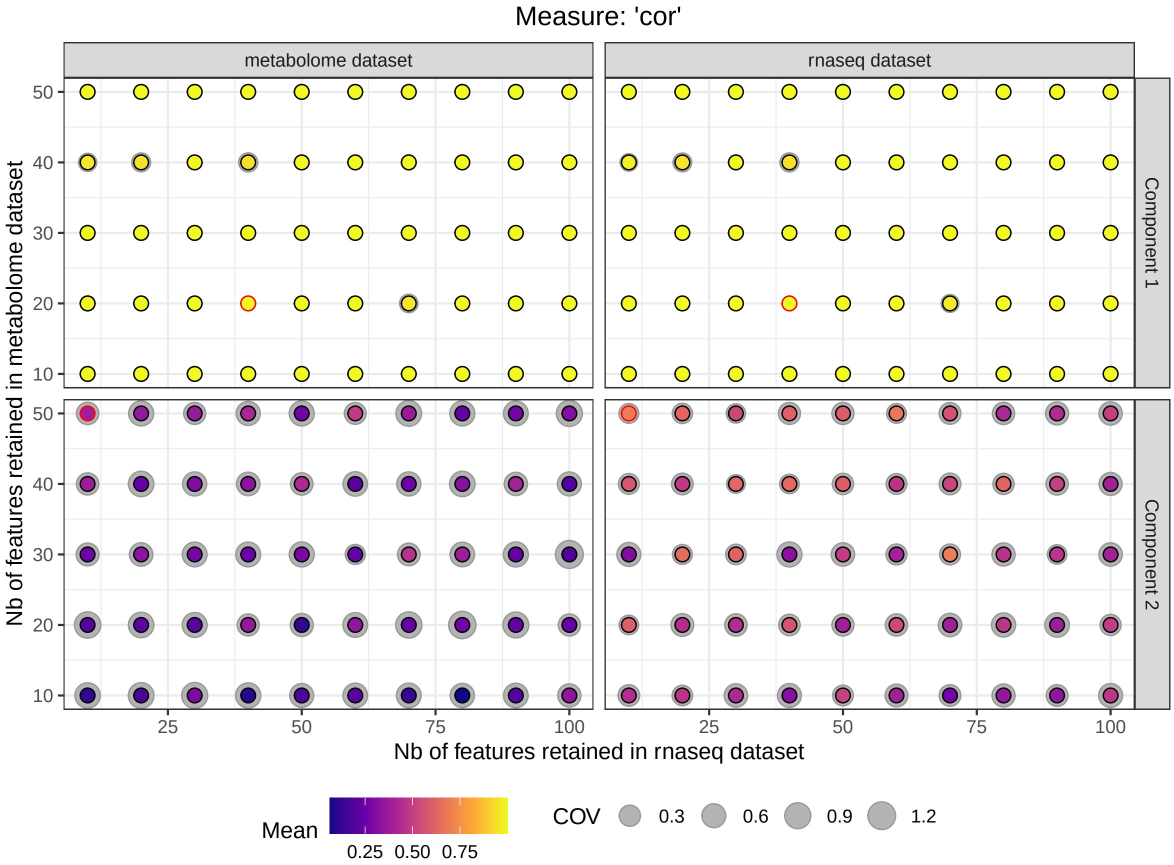

Figure 7: Cross-validation results for choosing the number of features to retain for each sPLS latent component. For each latent component (rows) and dataset (columns), the average correlation obtained across the cross-validation folds and repeats for each combination of values tested is shown by the colour of the points; the correlations' coefficient of variation calculated across the cross-validation folds and repeat is indicated by the size of the shaded area around the points.

Final sPLS run

Finally, sPLS is run using the selected values for the number of components to construct and the number of features to retain for each of these components.

Multi-omics integration with sO2PLS

The transformed and pre-filtered multi-omics dataset is used to generate an input object for sPLS; the transcriptomics and metabolomics datasets are selected for the analysis. moiraine automatically removes any samples that are not present across all omics datasets, and centres the features in each omics dataset.

Choosing the number of latent components

To select the number of joint and dataset-specific components to construct, an adjusted 10-fold cross-validation is first run. Values from 1 to 5 are tested for the number of joint components n, and values from 0 to 10, by increment of 2, are tested for the number of dataset-specific components n_x and n_y (corresponding to the trnascriptomics- and metabolomics-specific components, respectively). Briefly, the procedure estimates, for each possible value of n, the values of n_x and n_y that minimise the prediction error (MSE) for the joint part of each dataset1. Using these values, it then computes the prediction error for the full dataset decomposition and uses it to select the optimal value of n. The selected values are $n = 1$, $n_x = 0$ and $n_y = 4$ (Figure 8).

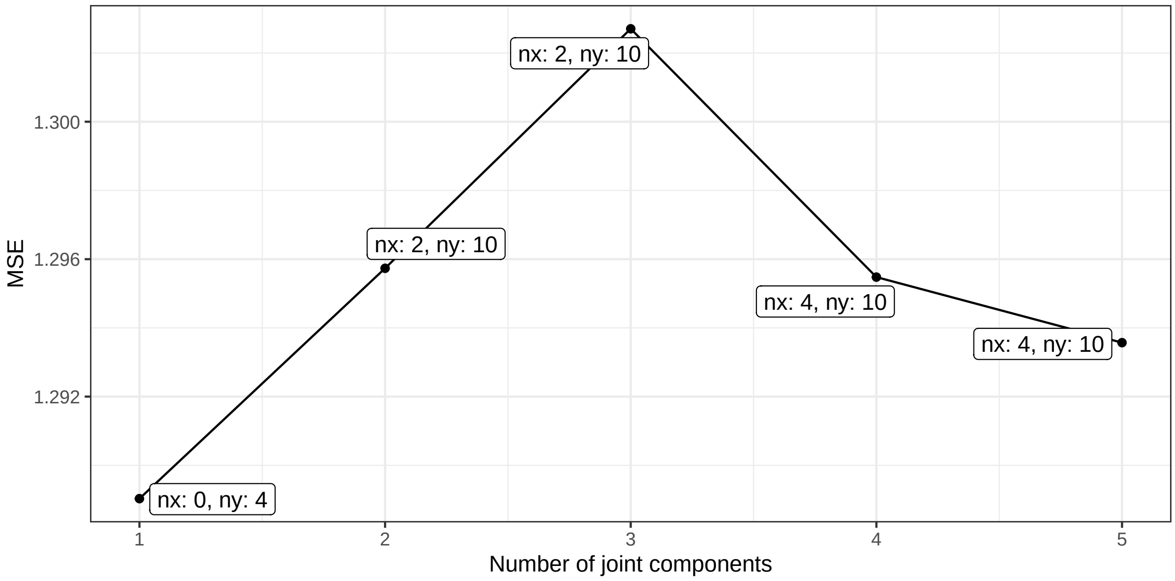

Figure 8: Adjusted cross-validation results for choosing the number of sO2PLS joint (x-axis) and dataset-specific latent components (labels) to construct.

In a second step, a standard 10-fold cross-validation is run; the tested values are as follows: $n = 1, 2$, $n_x = 0, 1$ and $n_y = 3, 4, 5$. The results are displayed in Figure 9; the final values retained are $n = 1$, $n_x = 1$, $n_y = 5$.

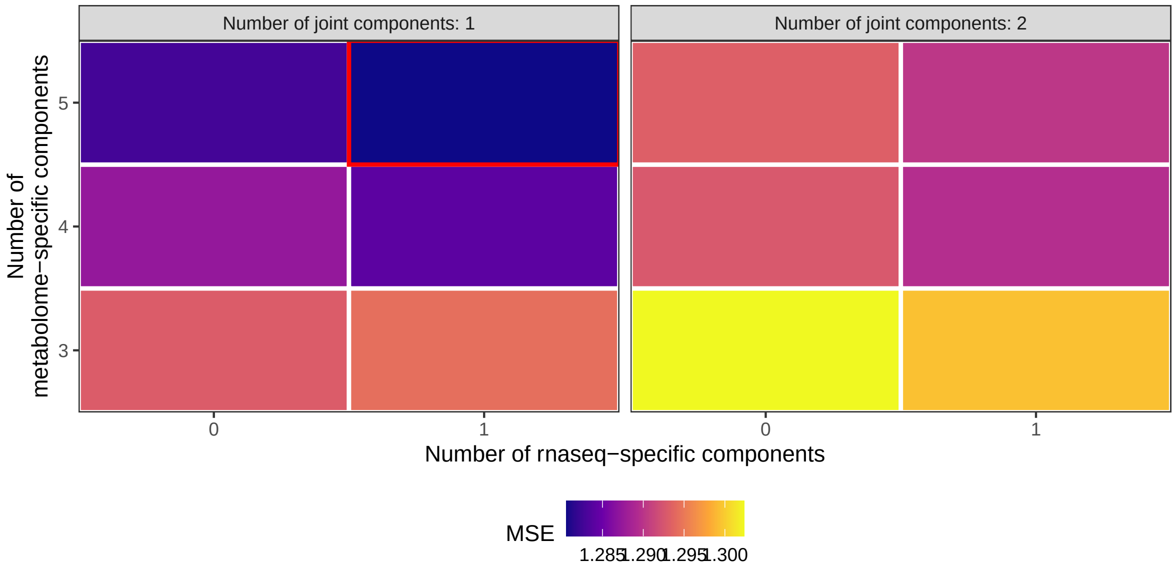

Figure 9: Standard cross-validation results for choosing the number of sO2PLS joint (facets) and dataset-specific latent components (x and y axes) to construct.

Choosing the number of features to retain

The optimal number of features to retain from each dataset for the joint components is selected via 10-fold cross-validation. Values from 5 to 30 by increment of 5 and from 40 to 100 by increment of 10 are tested for the number of transcripts, and values from 5 to 30 by increment of 5 are test for the number of metabolic compounds. The optimal combination of values is selected using the 1 standard deviation (SD) rule, i.e. select the lowest number of features for which the covariance between the joint components is within 1 SD of the maximum covariance obtained. The cross-validation results are shown in Figure 10, and the selected values in Table 2.

Table 2: Optimal number of features to retain from each omics dataset for the sO2PLS joint component, selected via cross-validation.

dataset	Joint component 1	Total
rnaseq	100	100
metabolome	25	25

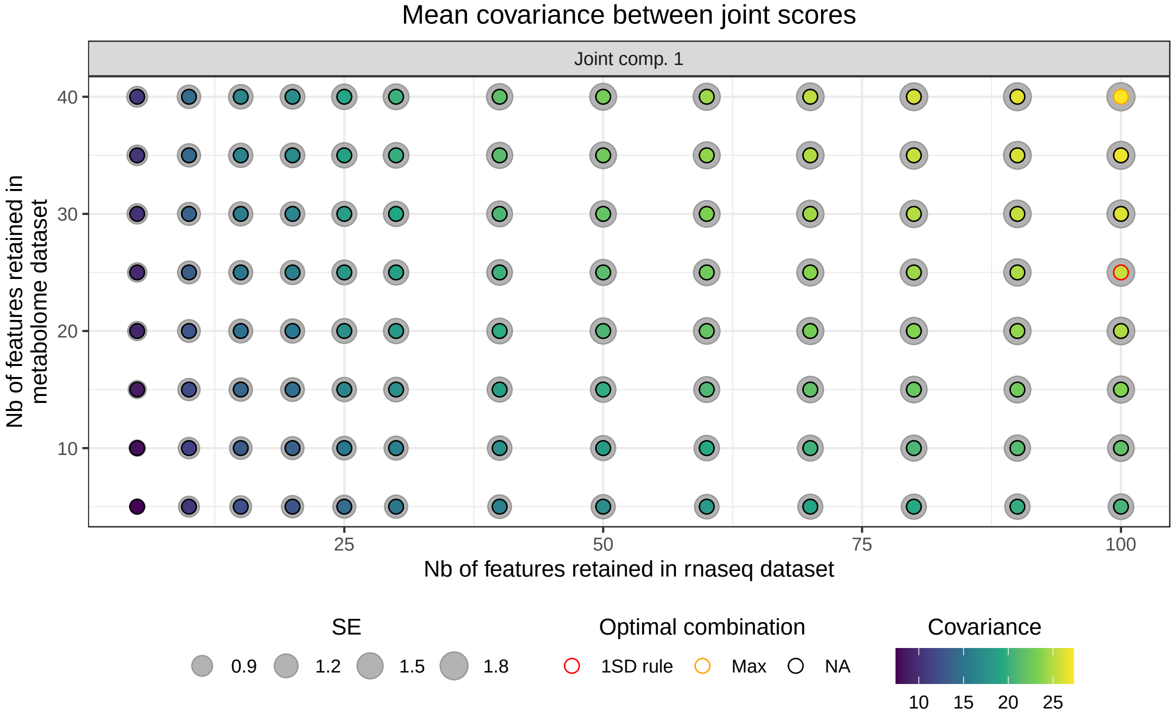

Figure 10: Cross-validation results for choosing the number of features to retain for the sO2PLS joint component.

Final sO2PLS run

Finally, sO2PLS is run using the selected values for the number of components to construct and the number of features to retain for each of these components.

Multi-omics integration with MOFA

The transformed and pre-filtered multi-omics dataset is used to generate an input object for MOFA. All samples are retained, even those missing from one or two omics layers; and the

datasets are scaled to unit variance. To train the model, a poisson likelihood is used for the genomics dataset, and a gaussian likelihood is used for the transcriptomics and metabolomics datasets.

Multi-omics integration with DIABLO

The transformed and pre-filtered multi-omics dataset is used to generate an input object for DIABLO. moiraine automatically removes any samples that are not present across all omics datasets. The disease status of the animals (i.e. control or infected) is used as sample outcome group.

Constructing the design matrix

A projection to latent structures (PLS) analysis is run on each pair of omics datasets; for a given pair, the correlation between the first latent component constructed for each dataset is used as an estimate of the correlation between the two datasets. These values are used to construct a design matrix for the DIABLO analysis. This matrix contains one row and one column for each omics dataset, plus an additional row and column for the samples outcome. Values within the matrix indicate the ration between the two optimisation objectives of DIABLO: maximising the covariance between the omics datasets, and maximising the discrimination of the outcome groups. The design matrix is constructed by moiraine as follows: - the values in the row and in the column corresponding to the samples outcome are set to 1; - the values in cells corresponding to a given pair of datasets are set to 0.1 if the estimated correlation between the pair of datasets is below 0.8, and to 1 otherwise.

Choosing the number of latent components

The optimal number of latent components to construct is selected via 10-fold cross-validation, repeated 10 times. Values from 1 to 7 are tested; the optimal value is selected using the centroid distance to compute samples outcome prediction and the balanced error rate to compute classification error. Results indicate that constructing four latent components minimises optimises the separation of control and infected animals (Figure 11).

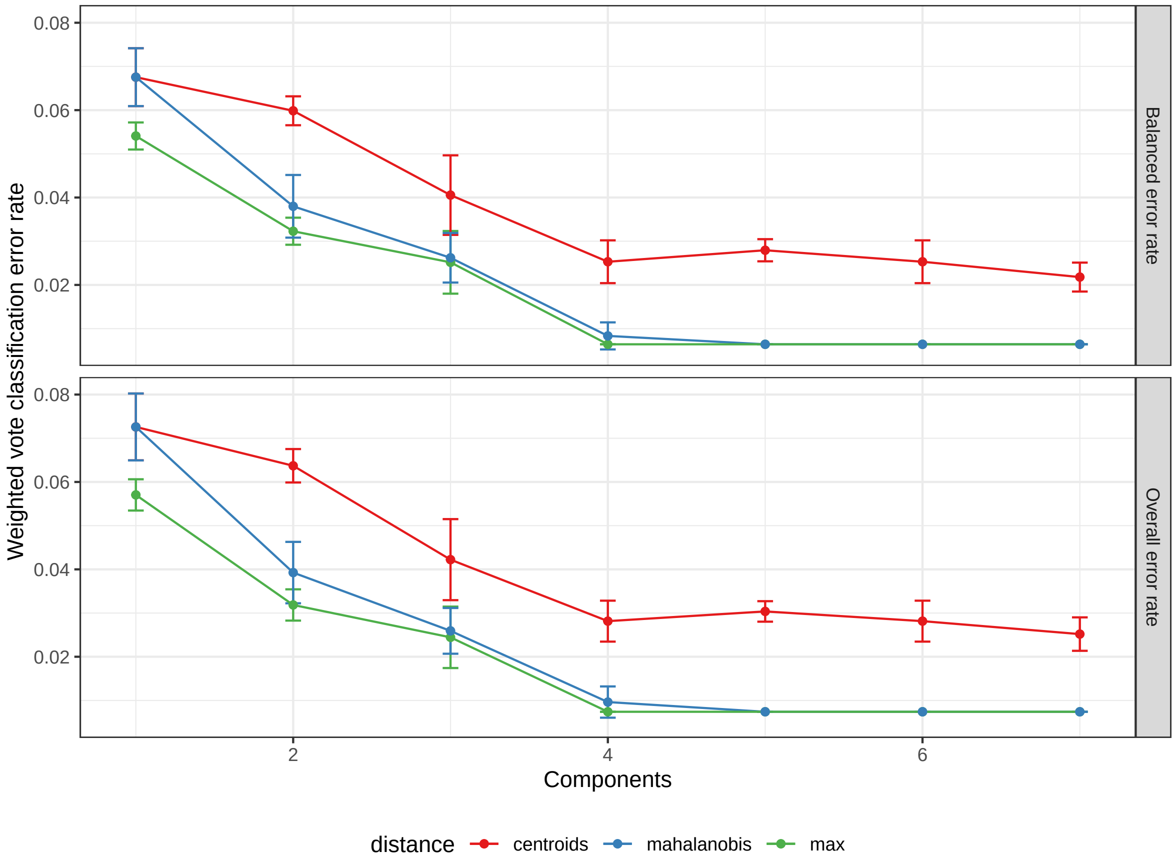

Figure 11: Cross-validation results for choosing the number of latent components to construct with DIABLO. The top and bottom panels depict two different metrics that can be used to assess classification error, namely the balanced error rate and overall error rate.

Choosing the number of features to retain

The optimal number of features to retain from each omics dataset for each of the four latent components to construct is selected via 10-fold cross-validation, repeated 5 times. Values from 5 to 30 by increment of 5 are tested; the optimal combination of values for each latent component is selected using the centroid distance to compute samples outcome prediction and the balanced error rate to compute classification error (Figure 12). The values selected are shown in Table 3.

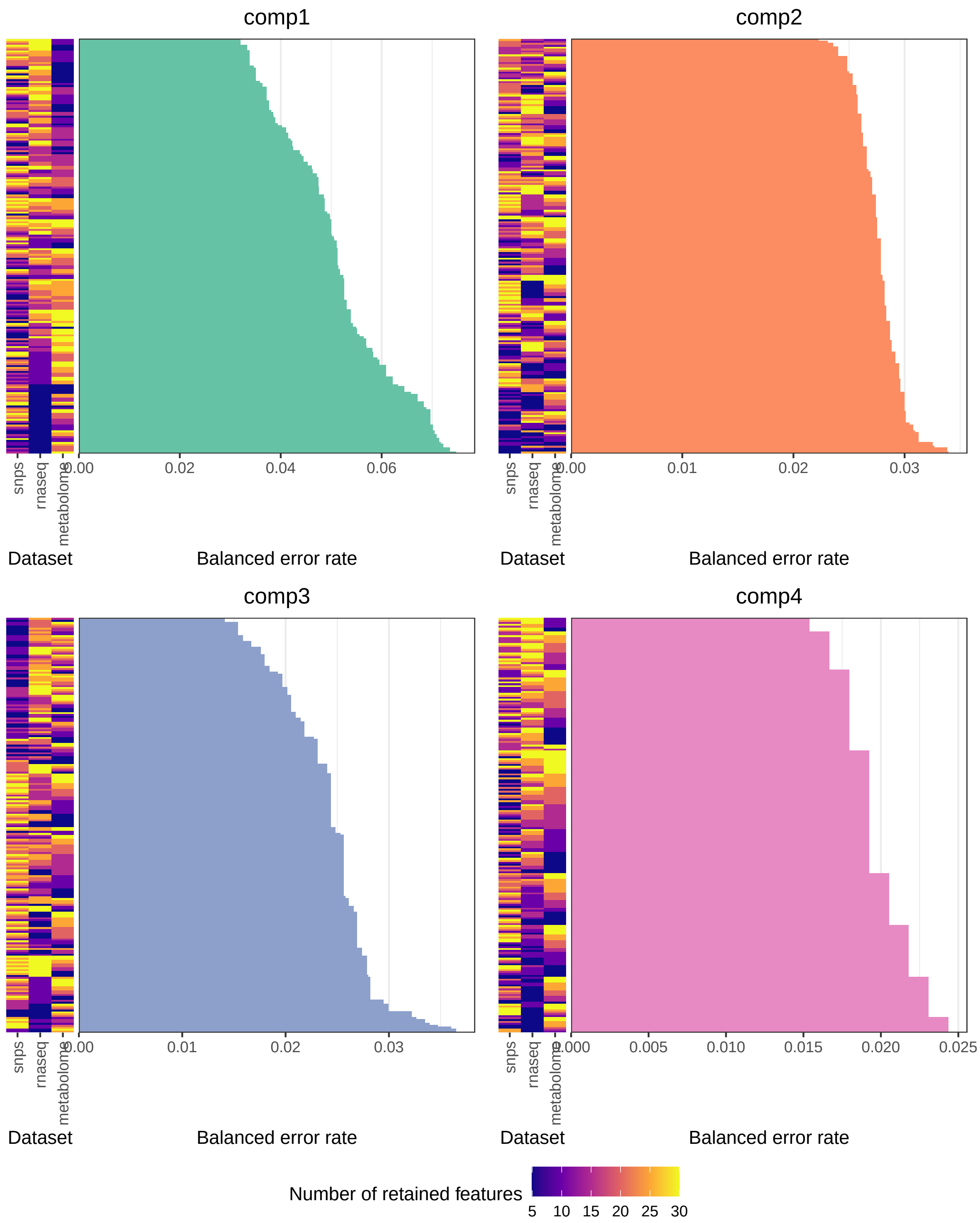

Figure 12: Cross-validation results for choosing the number of features to retain for each DIABLO latent component. For each latent component (panel), the combination of values tested are arranged by increasing balanced error rate.

Table 3: Optimal number of features to retain from each omics dataset for each DIABLO latent component, selected via cross-validation.

Dataset	Component 1	Component 2	Component 3	Component 4	Total
snps	20	25	5	25	75
rnaseq	30	15	20	30	95
metabolome	10	10	5	5	30

Final DIABLO run

Finally, DIABLO is run using the selected values for the number of components to construct and the number of features to retain for each of these components.

EATRIS-Plus dataset

The EATRIS-Plus project (Alonso-Andrés et al. 2023), funded by the European Commission Horizon 2020 Research programme, aimed at providing a high-quality multi-omics dataset from a cohort of healthy patients, to be used in precision medicine studies. To this aim, 127 healthy individuals from the Czech Genome Project were recruited, and measurements from the following omics layers were collected: mRNAseq, miRNAseq, miRNAseq (qRT-PCR), proteomics, enzymatic methylation sequencing, lipidomics from two ionisation modes, as well as three targeted metabolomics datasets (acylcaritines, amino acids, very long chain fatty acids). In addition, a number of clinical variables are recorded for the patients, including age, sex, smoking status, blood type, BMI, and measurements such as leukocytes or hemoglobin levels. The pre-processed multi-omics dataset is publicly available on Zenodo ('t Hoen and de Visser 2024) as a `MultiAssayExperiment` object.

For this analysis, we used the following omics layers:

- mRNAseq (19,747 features) – batch-adjusted and corrected values;
- miRNAseq (2,130 features) – batch- and cell type-adjusted values;
- proteomics (1,393 features) – values with missing values imputed;
- enzymatic methylation sequencing (100,000 features) – cell-type adjusted values for the 100,000 most variables CpG sites;
- lipidomics from positive ionisation mode (196 features): transformed values;
- lipidomics from negative ionisation mode (164 features): transformed values;
- Acylcarnitines (33 features) – batch-adjusted values with missing values imputed;
- Amino acids (46 features) – batch-adjusted values with missing values imputed.

Analysis of the EATRIS-Plus dataset

We give a brief overview of the analysis of the EATRIS-Plus dataset with moiraine. After importing the multi-omics dataset, the proteomics and two lipidomics datasets were centered and scaled. The RNAseq, miRNAseq, proteomics and enzymatic methylation datasets were then pre-filtered using sPLS-DA to retain at most 1,000 features, using patients sex as the outcome grouping variable. Sex was also used as the grouping variable for integration with DIABLO. 10-fold cross-validation repeated 10 times to assess the optimal number of latent components to construct. Values from 1 to 4 were tested; 3 was selected as the optimal value. Next, 10-fold cross-validation repeated 5 times was used to select the optimal number of features to retain for each latent component. The values tested were 10 and 30 for each omics

dataset and latent component. The multi-omics dataset was also integrated using MOFA; a Gaussian likelihood was used for all omics datasets.

Supplementary Figures

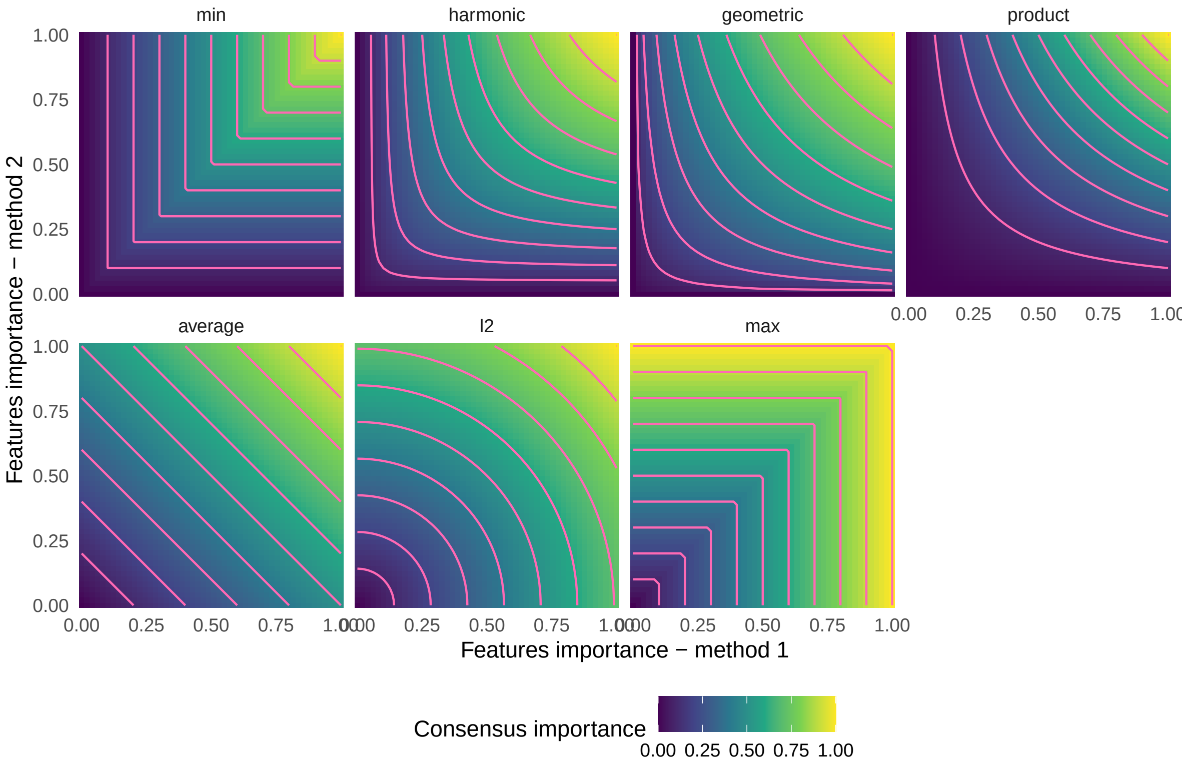

Figure S1: Overview of the consensus score obtained with different metrics when combining the importance score obtained with two different methods (x and y axes). The pink lines delimitate values for the individual importance scores for which the resulting consensus importance score is identical.

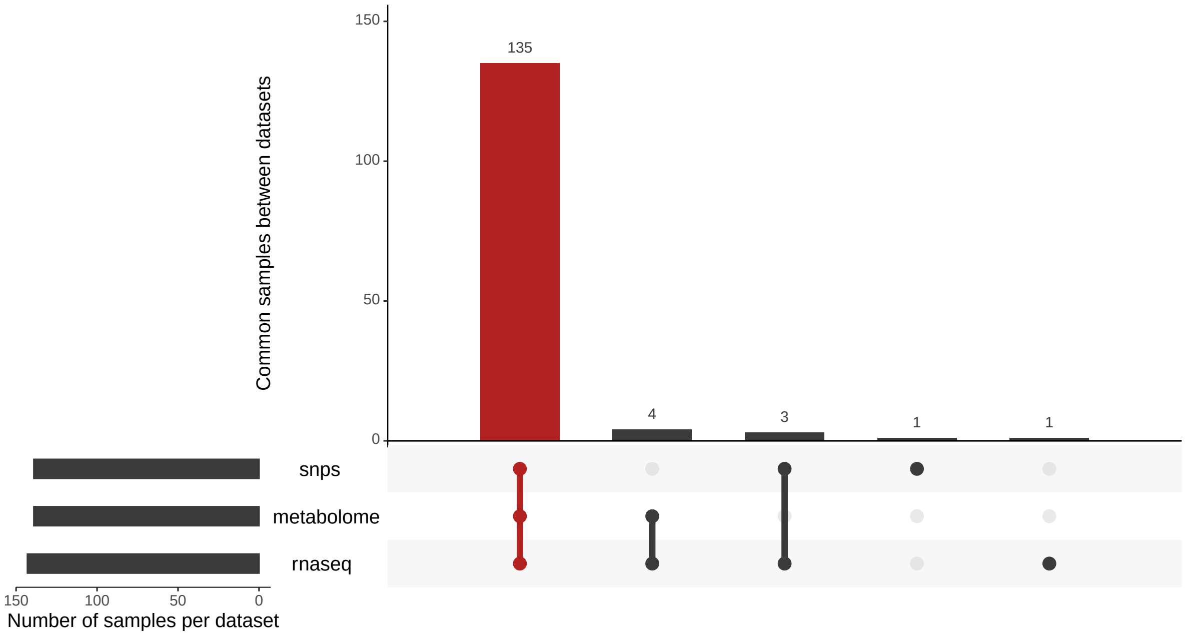

Figure S2: Upset plot comparing the samples present in the different omics datasets in the example data. Each row corresponds to an omics dataset; each column represents a unique subset of omics datasets, with the coloured dots joined by a line indicating which dataset is part of the subset. The horizontal bars show the number of samples present in each omics dataset; the vertical bars denote the number of samples that are present in all omics datasets that are part of the corresponding subset, and absent from the omics datasets not part of the subset. The column representing samples present in all three omics datasets is highlighted in red.

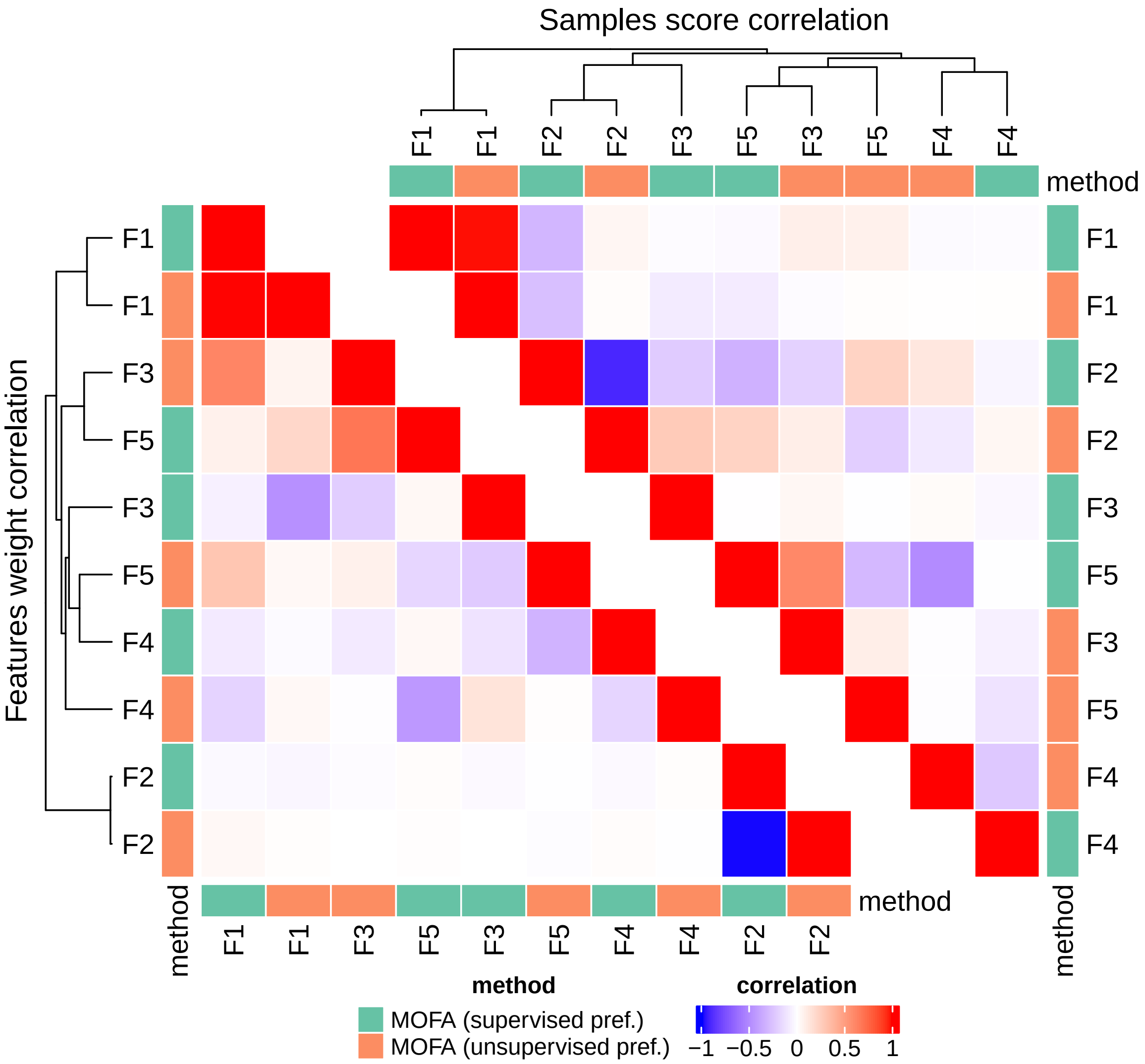

Figure S3: Correlation between the samples score (top-right) and features weight (bottom-left) of the first five latent factors constructed with MOFA after supervised pre-filtering or after unsupervised pre-filtering of the genomics and transcriptomics dataset.

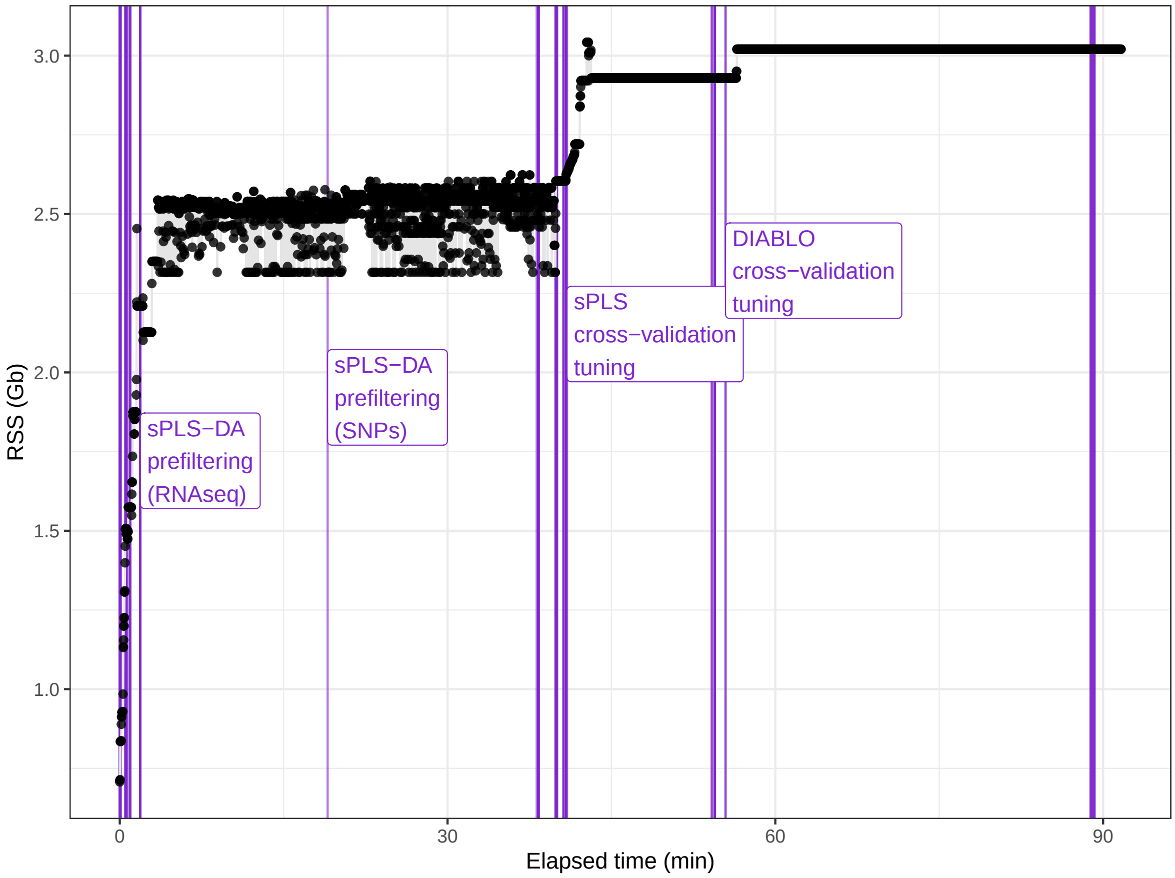

Figure S4: Resource usage of the analysis pipeline. The black dots indicate the resident set size (RSS – total amount of memory used) used over time, recorded every second. The purple vertical lines indicate the time at which the different steps in the pipeline (targets) are executed. The name of the longest steps is indicated.

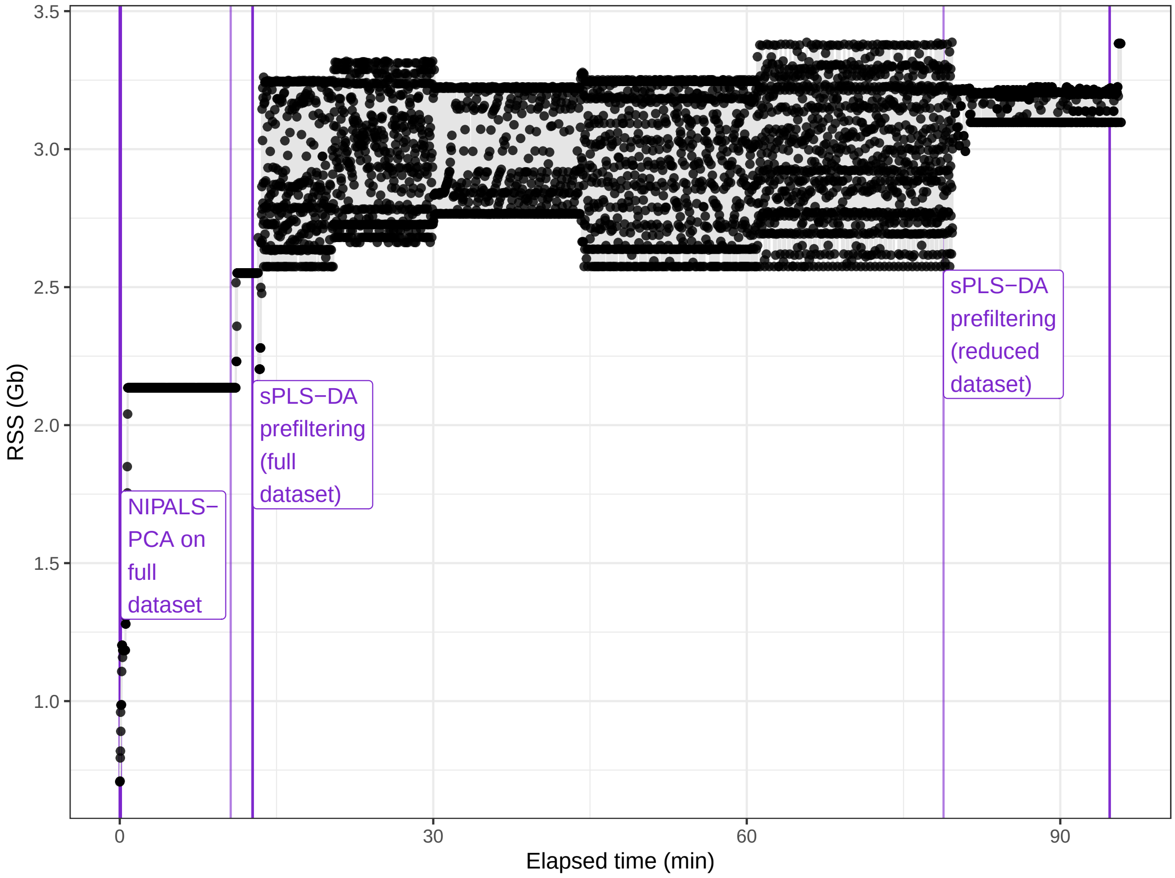

Figure S5: Comparison of resource usage for pre-processing the reduced genomics dataset used in the example (with 23,036 SNPs) vs the full genomics dataset (with 85,630 SNPs). The black dots indicate the resident set size (RSS – total amount of memory used) used over time, recorded every second. The purple vertical lines indicate the time at which the different steps in the pipeline (targets) are executed. The name of the longest steps is indicated.

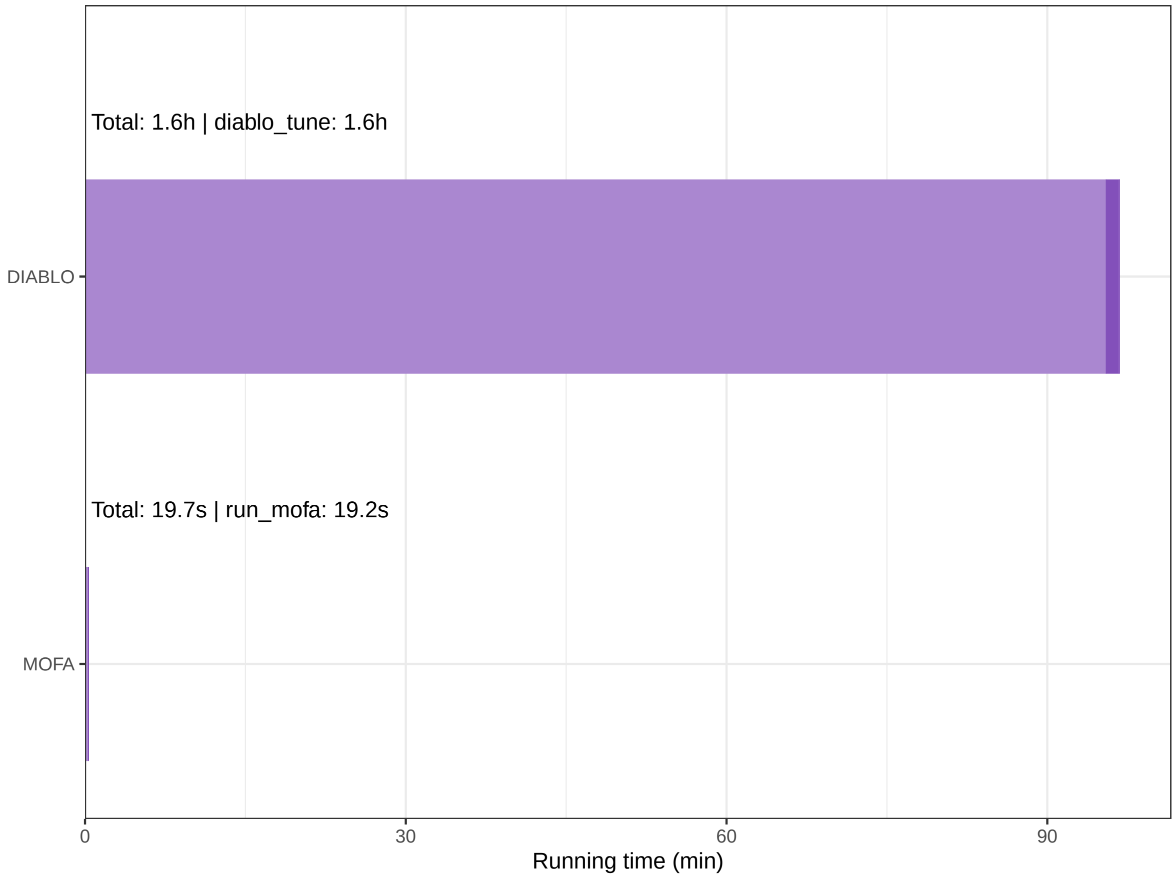

Figure S6: Running time of two integration tools (DIABLO and MOFA) on the EATRIS-Plus multi-omics dataset that comprises 8 different omics layers.

References

Alonso-Andrés, Patricia, Davide Baldazzi, Qiaochu Chen, Elisa Conde Moreno, Lorena Crespo-Toro, Kati Donner, Petr Džubák, et al. 2023. "Multi-Omics Quality Assessment in Personalized Medicine Through EATRIS." bioRxiv. https://doi.org/10.1101/2023.10.25.563912.

Argelaguet, Ricard, Britta Velten, Damien Arnol, Sascha Dietrich, Thorsten Zenz, John C Marioni, Florian Buettner, Wolfgang Huber, and Oliver Stegle. 2018. "Multi-Omics Factor Analysis—a Framework for Unsupervised Integration of Multi-Omics Data Sets." *Molecular Systems Biology* 14 (6): e8124. https://doi.org/10.15252/msb.20178124.

Bouhaddani, Said el, Hae-Won Uh, Geurt Jongbloed, Caroline Hayward, Lucija Klarić, Szymon M. Kiełbasa, and Jeanine Houwing-Duistermaat. 2018. "Integrating Omics Datasets with the OmicsPLS Package." *BMC Bioinformatics* 19 (1): 371. https://doi.org/10.1186/s12859-018-2371-3.

Cao, Kim-Anh Lê, Debra Rossouw, Christèle Robert-Granié, and Philippe Besse. 2008. "A Sparse PLS for Variable Selection When Integrating Omics Data." *Statistical Applications in Genetics and Molecular Biology* 7 (1). https://doi.org/10.2202/1544-6115.1390.

Hernandez-Ferrer, Carles, Carlos Ruiz-Arenas, Alba Beltran-Gomila, and Juan R. González. 2017. "MultiDataSet: An R Package for Encapsulating Multiple Data Sets with Application to Omic Data Integration." *BMC Bioinformatics* 18 (1): 36. https://doi.org/10.1186/s12859-016-1455-1.

Li, Jiyuan, Robert Mukiibi, Janelle Jiminez, Zhiquan Wang, Everestus C. Akanno, Edouard Timsit, and Graham S. Plastow. 2022. "Applying Multi-Omics Data to Study the Genetic Background of Bovine Respiratory Disease Infection in Feedlot Crossbred Cattle." *Frontiers in Genetics* 13.

Singh, Amrit, Casey P Shannon, Benoît Gautier, Florian Rohart, Michaël Vacher, Scott J Tebbutt, and Kim-Anh Lê Cao. 2019. "DIABLO: An Integrative Approach for Identifying Key Molecular Drivers from Multi-Omics Assays." *Bioinformatics* 35 (17): 3055–62. https://doi.org/10.1093/bioinformatics/bty1054.

't Hoen, Peter-Bram, and Casper de Visser. 2024. "EATRIS-Plus Multi-Omics Data of a Human Reference Cohort." Zenodo.

Velten, Britta, Jana M. Braunger, Ricard Argelaguet, Damien Arnol, Jakob Wirbel, Danila Bredikhin, Georg Zeller, and Oliver Stegle. 2022. "Identifying Temporal and Spatial Patterns of Variation from Multimodal Data Using MEFISTO." *Nature Methods* 19 (2): 179–86. https://doi.org/10.1038/s41592-021-01343-9.
