# Supplementary material for: moiraine: an R package to construct reproducible pipelines for the application and comparison of multi-omics integration methods
Source: Bioinformatics. 2026 Feb 15;42(3):btag070. doi: 10.1093/bioinformatics/btag070 (PMC12960915; doi:10.1093/bioinformatics/btag070)
Supplement: btag070_Supplementary_Data [file btag070_supplementary_data.zip › moiraine_publication_code-main.zip!/moiraine_publication_code-main/output/figure_methods_comparison.pdf]

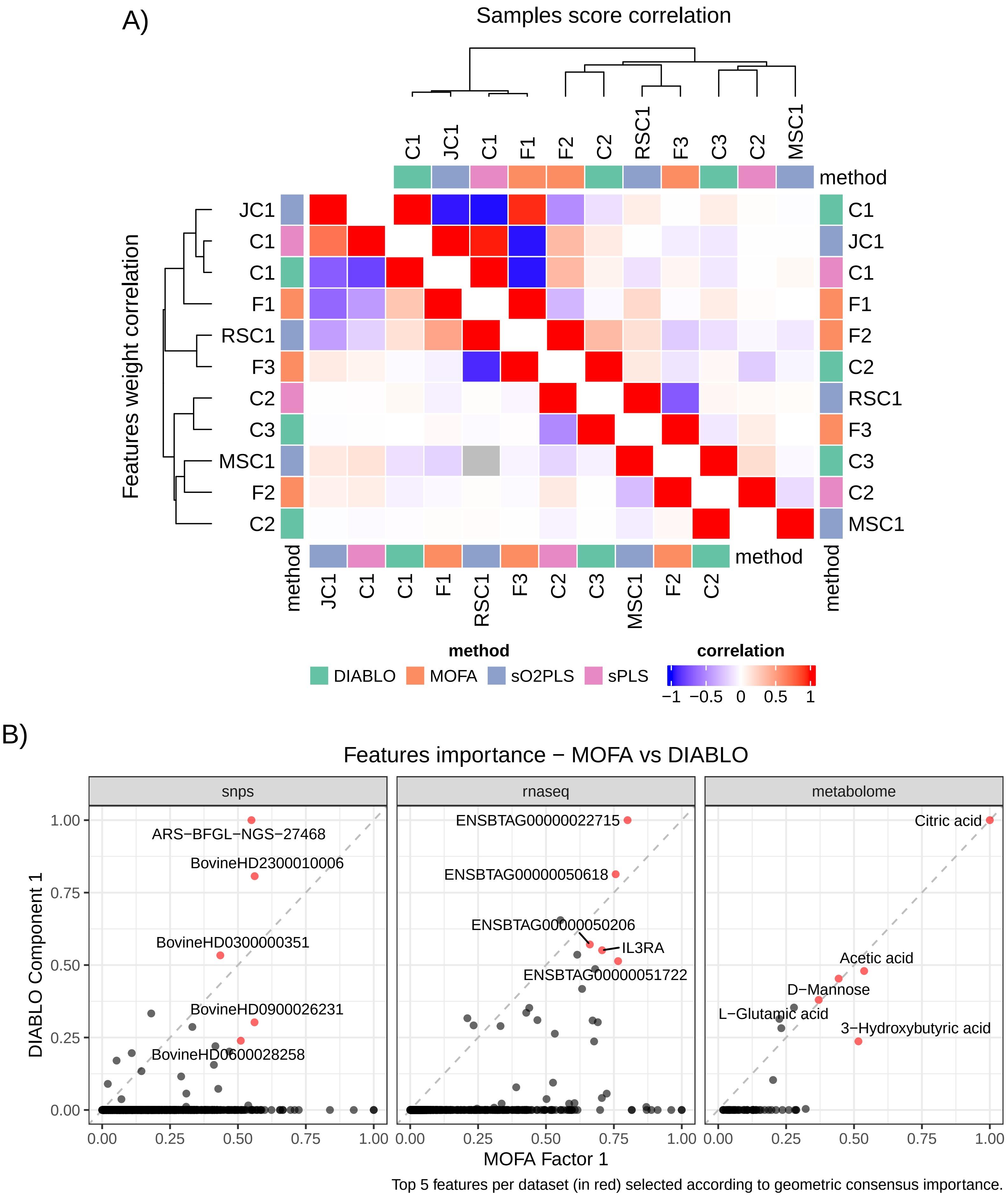
A)
Samples score correlation
Features weight correlation
method
JC1
C1
C1
F1
RSC1
F3
C2
C3
MSC1
F2
C2
C1
JC1
C1
F1
F2
C2
RSC1
F3
C3
C2
MSC1
method
DIABLO
MOFA
sO2PLS
sPLS
correlation
−1
−0.5
0
0.5
1
B)
Features importance − MOFA vs DIABLO
snps
rnaseq
metabolome
ARS−BFGL−NGS−27468
BovineHD2300010006
BovineHD0300000351
BovineHD0900026231
BovineHD0600028258
ENSBTAG00000022715
ENSBTAG00000050618
ENSBTAG00000050206
IL3RA
ENSBTAG00000051722
Citric acid
Acetic acid
D−Mannose
L−Glutamic acid
3−Hydroxybutyric acid
DIABLO Component 1
MOFA Factor 1
0.00
0.25
0.50
0.75
1.00
Top 5 features per dataset (in red) selected according to geometric consensus importance.
