# Supplementary material for: moiraine: an R package to construct reproducible pipelines for the application and comparison of multi-omics integration methods
Source: Bioinformatics. 2026 Feb 15;42(3):btag070. doi: 10.1093/bioinformatics/btag070 (PMC12960915; doi:10.1093/bioinformatics/btag070)
Supplement: btag070_Supplementary_Data [file btag070_supplementary_data.zip › moiraine_publication_code-main.zip!/moiraine_publication_code-main/output/figure_mofa_features.pdf]

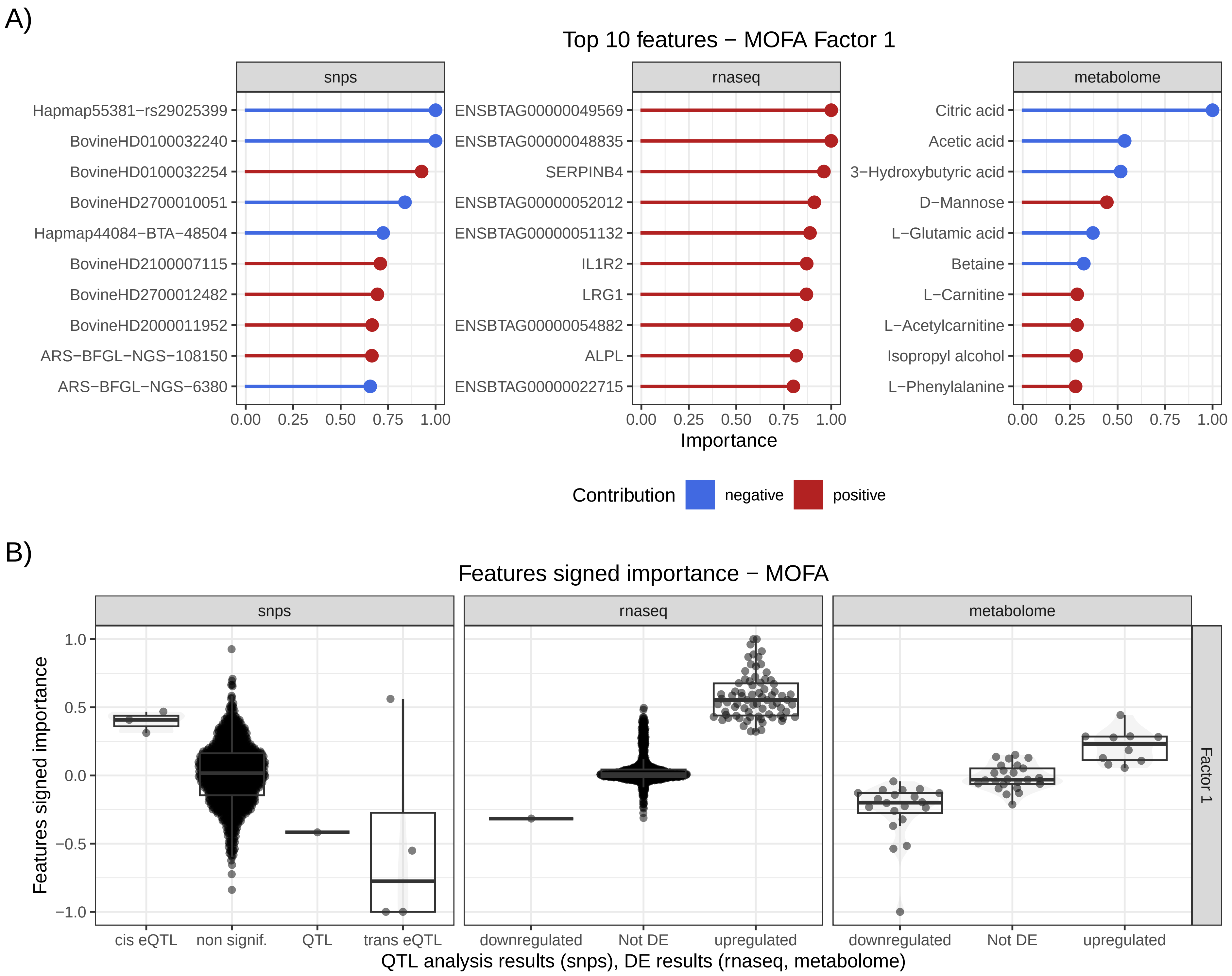
A)
Top 10 features − MOFA Factor 1
snps
rnaseq
metabolome
Hapmap55381−rs29025399
BovineHD0100032240
BovineHD0100032254
BovineHD2700010051
Hapmap44084−BTA−48504
BovineHD2100007115
BovineHD2700012482
BovineHD2000011952
ARS−BFGL−NGS−108150
ARS−BFGL−NGS−6380
ENSBTAG00000049569
ENSBTAG00000048835
SERPINB4
ENSBTAG00000052012
ENSBTAG00000051132
IL1R2
LRG1
ENSBTAG00000054882
ALPL
ENSBTAG00000022715
Citric acid
Acetic acid
3−Hydroxybutyric acid
D−Mannose
L−Glutamic acid
Betaine
L−Carnitine
L−Acetylcarnitine
Isopropyl alcohol
L−Phenylalanine
0.00 0.25 0.50 0.75 1.00
Importance
Contribution negative positive
B)
Features signed importance − MOFA
snps
rnaseq
metabolome
Factor 1
Features signed importance
1.0 0.5 0.0 −0.5 −1.0
cis eQTL
non signif.
QTL
trans eQTL
downregulated
Not DE
upregulated
downregulated
Not DE
upregulated
QTL analysis results (snps), DE results (rnaseq, metabolome)
