# Supplementary material for: moiraine: an R package to construct reproducible pipelines for the application and comparison of multi-omics integration methods
Source: Bioinformatics. 2026 Feb 15;42(3):btag070. doi: 10.1093/bioinformatics/btag070 (PMC12960915; doi:10.1093/bioinformatics/btag070)
Supplement: btag070_Supplementary_Data [file btag070_supplementary_data.zip › moiraine_publication_code-main.zip!/moiraine_publication_code-main/output/figure_pca_rnaseq.pdf]

A)

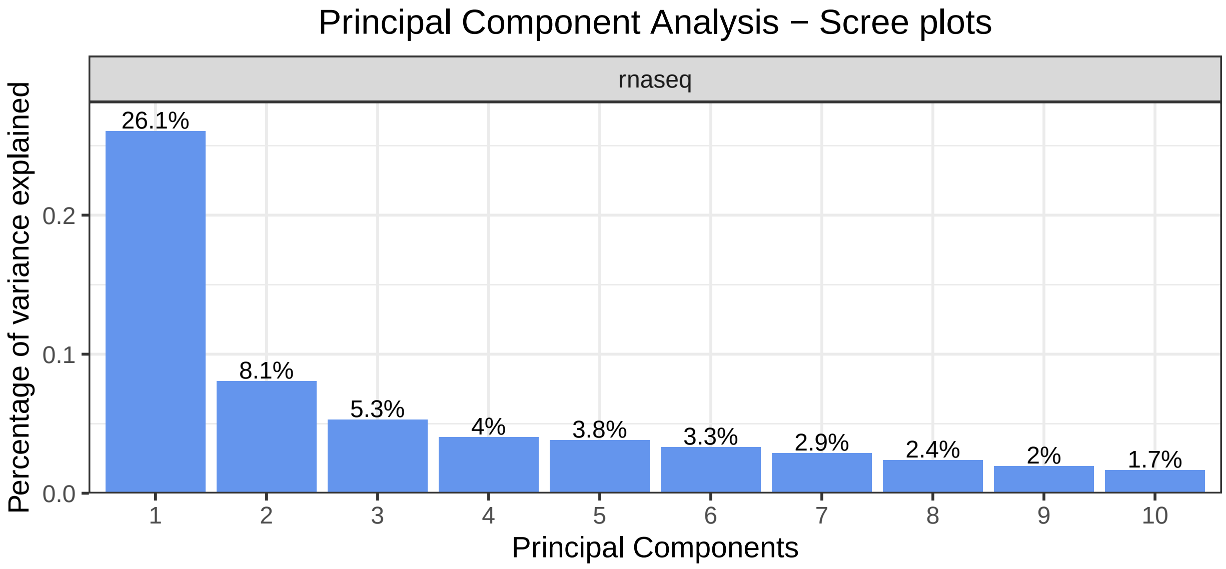
Principal Component Analysis − Scree plots
rnaseq
Percentage of variance explained
0.0
0.1
0.2
26.1%
8.1%
5.3%
4%
3.8%
3.3%
2.9%
2.4%
2%
1.7%
1
2
3
4
5
6
7
8
9
10
Principal Components

B)

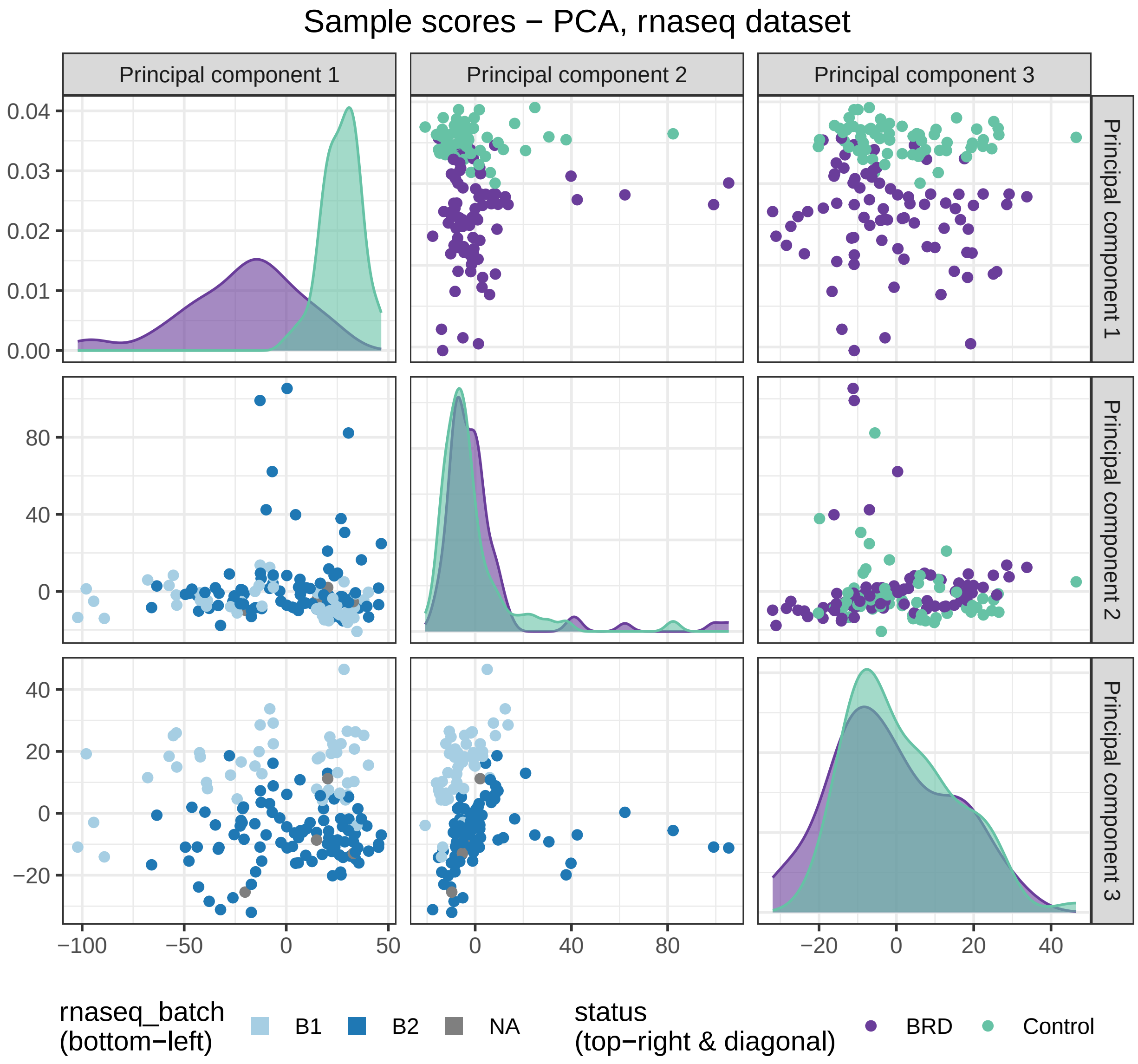
Sample scores − PCA, rnaseq dataset
Principal component 1
Principal component 2
Principal component 3
0.00
0.01
0.02
0.03
0.04
80
40
0
40
20
0
−20
−100
−50
0
50
0
40
80
−20
0
20
40
rnaseq_batch
(bottom−left)
B1
B2
NA
status
(top−right & diagonal)
BRD
Control
