# Supplementary material for: moiraine: an R package to construct reproducible pipelines for the application and comparison of multi-omics integration methods
Source: Bioinformatics. 2026 Feb 15;42(3):btag070. doi: 10.1093/bioinformatics/btag070 (PMC12960915; doi:10.1093/bioinformatics/btag070)
Supplement: btag070_Supplementary_Data [file btag070_supplementary_data.zip › moiraine_publication_code-main.zip!/moiraine_publication_code-main/output/figure_running_times.pdf]

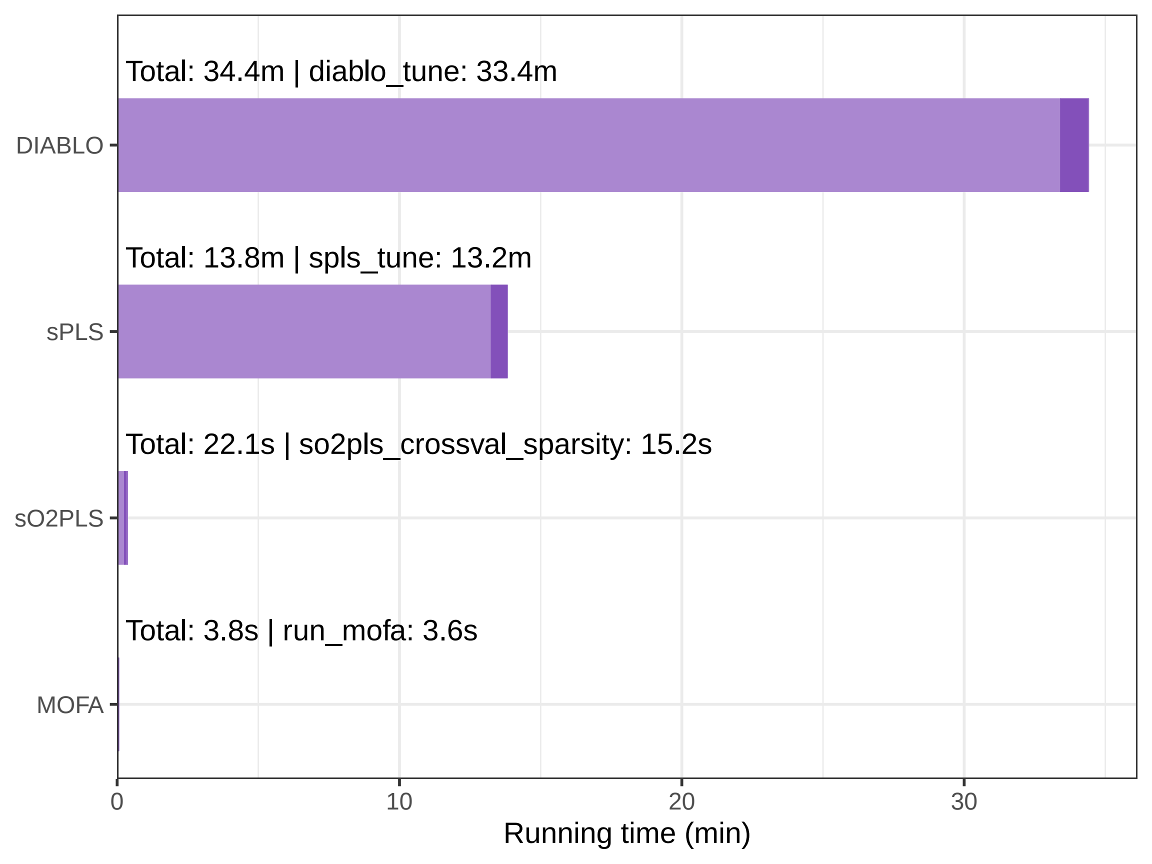
Total: 34.4m | diablo_tune: 33.4m
Total: 13.8m | spls_tune: 13.2m
Total: 22.1s | so2pls_crossval_sparsity: 15.2s
Total: 3.8s | run_mofa: 3.6s
DIABLO
sPLS
sO2PLS
MOFA
0
10
20
30
Running time (min)
