# Supplementary material for: moiraine: an R package to construct reproducible pipelines for the application and comparison of multi-omics integration methods
Source: Bioinformatics. 2026 Feb 15;42(3):btag070. doi: 10.1093/bioinformatics/btag070 (PMC12960915; doi:10.1093/bioinformatics/btag070)
Supplement: btag070_Supplementary_Data [file btag070_supplementary_data.zip › moiraine_publication_code-main.zip!/moiraine_publication_code-main/output/figure_samples_score_integration.pdf]

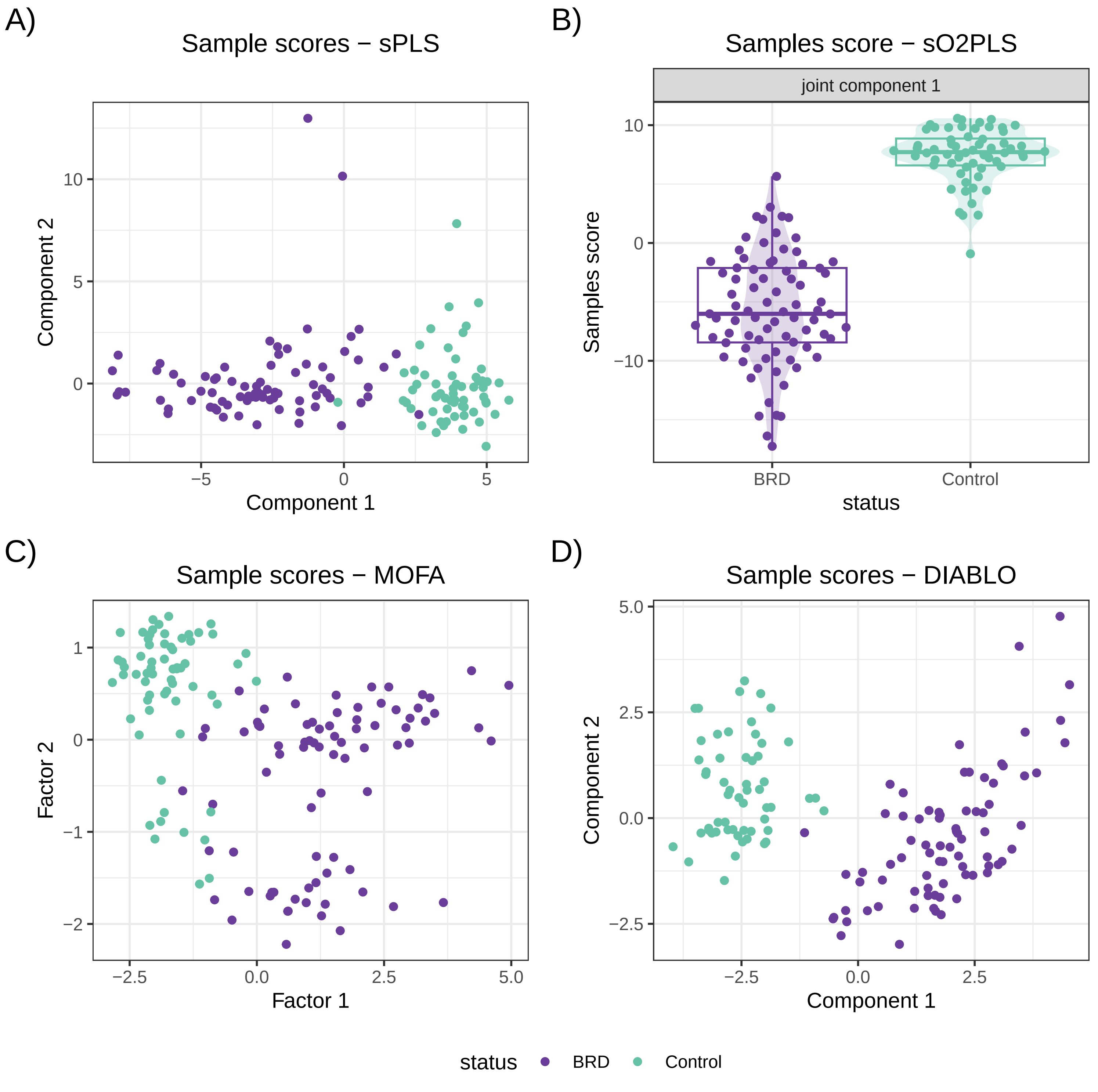

A)
Sample scores − sPLS
Component 2
Component 1
10
5
0
−5
0
5
B)
Samples score − sO2PLS
joint component 1
Samples score
10
0
−10
BRD
Control
status
C)
Sample scores − MOFA
Factor 2
Factor 1
1
0
−1
−2
−2.5
0.0
2.5
5.0
D)
Sample scores − DIABLO
Component 2
Component 1
5.0
2.5
0.0
−2.5
−2.5
0.0
2.5
status
BRD
Control
